# Tumor treating fields increases membrane permeability in glioblastoma cells

**DOI:** 10.1038/s41420-018-0130-x

**Published:** 2018-12-05

**Authors:** Edwin Chang, Chirag B. Patel, Christoph Pohling, Caroline Young, Jonathan Song, Thomas Anthony Flores, Yitian Zeng, Lydia-Marie Joubert, Hamed Arami, Arutselvan Natarajan, Robert Sinclair, Sanjiv S. Gambhir

**Affiliations:** 1 Molecular Imaging Program at Stanford, Department of Radiology, Stanford University School of Medicine, Room E150, 318 Campus Drive West, Stanford, CA 94305 USA; 2 Division of Neuro-Oncology, Department of Neurology and Neurological Sciences, Stanford University School of Medicine, Stanford, CA 94305 USA; 3 Department of Applied Physics, Stanford University School of Medicine, Stanford, CA 94305 USA; 4 Department of Materials Science & Engineering, Stanford University School of Medicine, Stanford, CA 94305 USA; 5 Electron Microscopy Unit, Stellenbosch University, Stellenbosch, South Africa; 6 Department of Bioengineering, Stanford University School of Medicine, Stanford, CA 94305 USA

## Abstract

Glioblastoma is the most common yet most lethal of primary brain cancers with a one-year post-diagnosis survival rate of 65% and a five-year survival rate of barely 5%. Recently the U.S. Food and Drug Administration approved a novel fourth approach (in addition to surgery, radiation therapy, and chemotherapy) to treating glioblastoma; namely, tumor treating fields (TTFields). TTFields involves the delivery of alternating electric fields to the tumor but its mechanisms of action are not fully understood. Current theories involve TTFields disrupting mitosis due to interference with proper mitotic spindle assembly. We show that TTFields also alters cellular membrane structure thus rendering it more permeant to chemotherapeutics. Increased membrane permeability through the imposition of TTFields was shown by several approaches. For example, increased permeability was indicated through increased bioluminescence with TTFields exposure or with the increased binding and ingress of membrane-associating reagents such as Dextran-FITC or ethidium D or with the demonstration by scanning electron microscopy of augmented number and sizes of holes on the cellular membrane. Further investigations showed that increases in bioluminescence and membrane hole production with TTFields exposure disappeared by 24 h after cessation of alternating electric fields thus demonstrating that this phenomenom is reversible. Preliminary investigations showed that TTFields did not induce membrane holes in normal human fibroblasts thus suggesting that the phenomenom was specific to cancer cells. With TTFields, we present evidence showing augmented membrane accessibility by compounds such as 5-aminolevulinic acid, a reagent used intraoperatively to delineate tumor from normal tissue in glioblastoma patients. In addition, this mechanism helps to explain previous reports of additive and synergistic effects between TTFields and other chemotherapies. These findings have implications for the design of combination therapies in glioblastoma and other cancers and may significantly alter standard of care strategies for these diseases.

## Background

Treatment of glioblastoma (GBM) by tumor treating fields (TTFields) is a novel, validated therapy that has become an additional modality (after surgery chemoradiation^1,2^ and chemotherapy) for anti-cancer treatments^3,4^. Originally studied in 1964 in human erythrocytes, distortions from high frequency electric fields (120 MHz) led to a reversible elongation accompanied by rotatory motions of cells^5^. Since those initial observations, intermediate frequency alternating electric fields (100–500 kHz), or TTFields, have been studied in detail^6–8^. Most recently, TTFields has been shown to prolong median survival (by 5 months) of glioblastoma patients on maintenance temozolomide chemotherapy^2,9^.

**Fig. 1:**
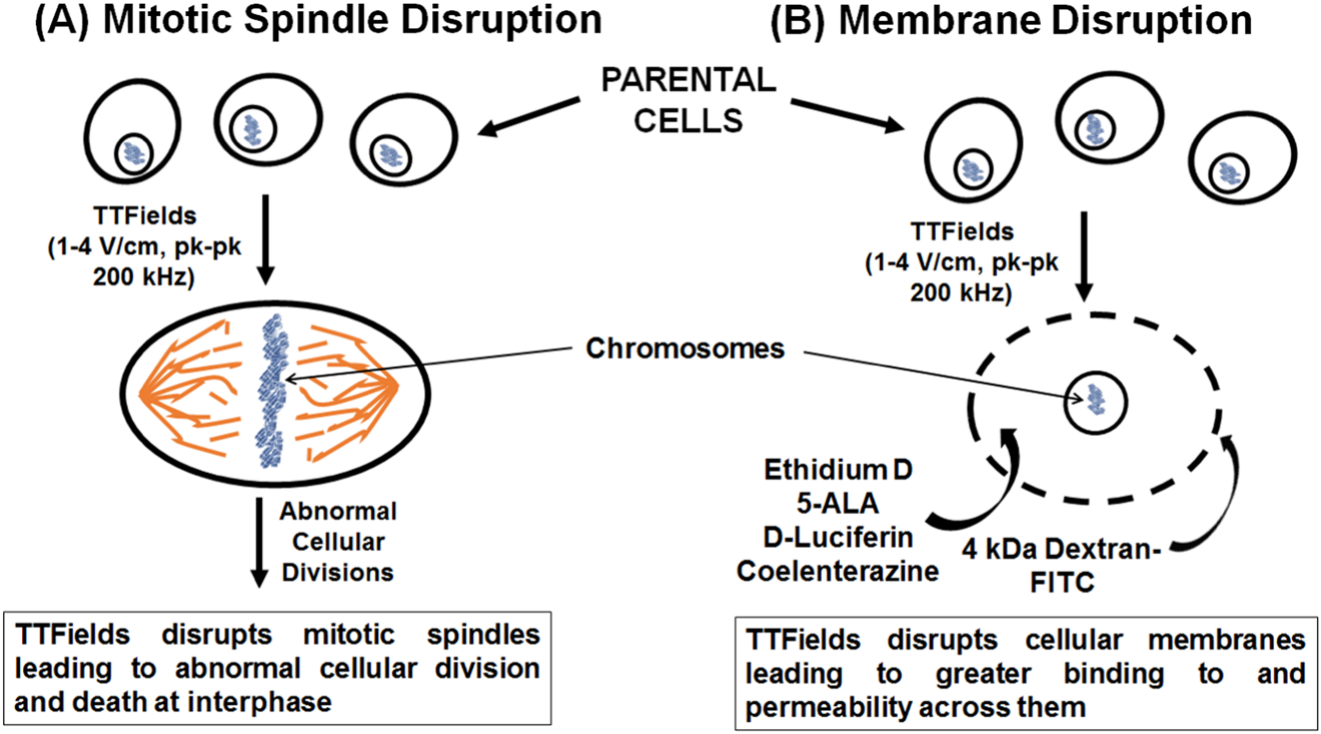
**a** Schematic showing classical view of the alteration of the mitotic spindle during mitosis by TTFields that results in cancer cell death. **b** Schematic showing an alternative effect of TTFields on modulating the integrity and thus the permeability of cancer cellular membranes. 5-ALA, 5-aminolevulinic acid, Ethidium D, ethidium bromide, FITC, fluorescein isothiocyanate, pk, peak, TTFields, tumor treating fields

The most widely proposed (“standard”) mechanism of anti-cancer action by TTFields centers upon the property that tubulin subunits have intrinsic dipole moments^8^. By forcing microtubule structures to align along alternating electric field lines through exogenous imposition of 200 kHz TTFields, the functionality of actively dividing cells is disrupted (Fig. 1a^10^) through interference with the cytoskeleton supporting mitotic spindles^7,8,11^. Such stress ulitmately promotes impaired cellular proliferation^7,8,11^. Proof of concept experiments and relevant technological developments have occurred over the past ten years^8,11^, culminating in the approval by the Food and Drug Administration (FDA) of a commercial, clinical TTFields device (Optune^®^, Novocure Ltd., Jersey, UK) in 2011 and 2015 for the treatment of recurrent and newly-diagnosed glioblastoma, respectively^2,9,12,13^.

More insights on mechanisms of action have been reported. TTFields has been shown to disrupt the localization of septins (intracellular proteins responsible for anchoring mitotic spindles during cellular division) and thereby perturb mitosis^14^. Some have reported prolongation of DNA damage by chemotherapy or radiotherapy^6,11,15^ in conjunction with TTFields while others have shown effects on mitochondrial function through the swelling of mitochondrial matrices^16^. Other teams explored combination of chemotherapies (e.g., temozolomide) with TTFields in GBM patients^2,9^. Such research into combination interventions has uncovered other promising effects against glioblastoma^6,17^.

Recently we have demonstrated that TTFields treatment, in conjunction with a novel anticancer compound Withaferin A, synergistically inhibited the growth of human glioblastoma cells^18^. We hypothesized that such a synergistic effect is due to increased accessibility of Withaferin A to glioblastoma cells through TTFields’ capability to increase transiently, tumor cell membrane permeability (Fig. 1b). In this study, we present data that validate the hypothesis. In particular, we provide evidence to show that TTFields exposure induced greater bioluminescence in human glioblastoma cells that have been modified to express luciferase (renilla and firefly), and that this induction is due to increased permeation of the substrates (d-luciferin and coelenterazine, respectively), through the plasma membrane. Increased membrane permeability caused by TTFields exposure is also demonstrated with other membrane-penetrating reagents such as Dextran-FITC and Ethidium D.

5-ALA is a hemoglobin precursor that is converted into fluorescent protoporphyrin IX (PpIX) in all mammalian cells^19^. Malignant cells, including high-grade gliomas, have elevated hemoglobin biosynthesis, reflected in enhanced accumulation of PpIX within transformed cells and tissues^20–22^. Medical investigations thus use 5-ALA uptake (and, by consequence, its enzymatic conversion to PpIX) as a fluorescent biomarker for tumor cells^20,22^. With current technologies, it is difficult to distinguish the precise cellular margin between tumor and non-tumor tissue intraoperatively^23,24^. We show that TTFields significantly enhances the tumor to normal cell ratio for PpIX fluorescence (brought on by 5-ALA exposure and uptake), and in this manner, may better delineate tumor margins in intraoperative settings.

Finally, we present scanning electron microscopy (SEM) data that demonstrate an increase in the number and size of holes in glioblastoma cell membranes caused by TTFields exposure. Furthermore, we show that the morphology of the glioblastoma cell membrane is perturbed when TTFields are applied. Through all modalities studied (bioluminescence, fluorescence, and SEM), we found the effects of TTFields on the GBM cell membrane permeability to be reversible after cessation of TTFields exposure.

## Material and methods

### Cell culture studies

Two patient-derived GBM lines (GBM2^25,26^, GBM39^27,28^), a commercially available human GBM cell line (U87-MG from ATCC, Manassas, VA, USA) as well as a murine astrocytoma cell line, (KR158B; a gift from Dr. Duane Mitchell of the Department of Neurosurgery at the University of Florida School of Medicine) were used for our studies.

Human U87-MG, human PCS-201 and murine KR158B glioblastoma cell lines were grown in DMEM (Invitrogen/Life Technologies, Carlsbad, CA, USA)/10% FBS/ and 1× antibiotic-antimycotic (Invitrogen/Life Technologies, Carlsbad, CA). GBM2 and GBM39 were grown in a defined, serum-free media whose composition has been described previously^18^.

### Seeding of cells onto glass coverslips for TTFields experiments

Briefly, cells in culture were trypsinized via standard protocols^26,29^ and 10,000–50,000 single cells were suspended in 200 or 75 µL of DMEM/10% FBS/1× antibiotic-antimycotic and then were seeded onto the center of a 22 mm or 12 mm diameter glass Thermanox^TM^ coverslips respectively (ThermoFisher Scientific, Waltham, MA, USA). The cells were incubated overnight in a humidified 95% air/5% CO_2_ incubator set at 37 ^o^C. Once the cells became attached to the coverslip, 2 mL or 1 mL of DMEM/10% FBS/1× antibiotic-antimycotic was added per well of 6-well or 12-well plates, respectively. Unless otherwise stated in the Results section, the cells were left to grow on the coverslip for two to three days (in order to ensure cells were in the growth phase) before being transferred to ceramic dishes of an inovitro^TM^ in vitro TTFields apparatus (Novocure Inc., Haifa, Israel). Growth conditions (i.e., time cells allowed to grow under TTFields-exposed vs. unexposed conditions) are specified either in the Results section or in the corresponding figure legends.

### In vitro tumor treating field apparatus^30^

The coverslips were transferred to a ceramic dish of the inovitro^TM^ system, which in turn was mounted onto inovitro^TM^ base plates (Novocure Ltd., Haifa, Israel). Tumor treating fields at 200 kHz (1–4 V/cm) were applied through an inovitro^TM^ power generator. Incubator ambient temperatures spanned 20–27 ^o^C with a target temperature of 37 ^o^C in the ceramic dishes upon application of the TTFields. Duration of TTFields exposure lasted anywhere from 0.5 to 72 h, after which coverslips were removed and processed for the appropriate bioassays (see below). For reversibility experiments, the TTFields-exposed coverslips were transferred to a regular incubator without TTFields exposure for 24 h (off TTFields period to assess for reversibility of the TTFields effect on cell membrane permeability) prior to processing for the appropriate bioassays. Culture media were exchanged manually every 24 h throughout the experiments to account for evaporation. Corresponding control experiments (no TTFields) were done by placing equivalent coverslips within 6-well or 12-well plates into a conventional humidified tissue culture incubator (37 ^o^C, 95% air/5% CO_2_) and cells grown in parallel with the TTFields-exposed coverslips. Unless otherwise mentioned, all experiments were done in at least triplicate samples per condition and per time point. A basic workflow for a typical TTFields experiment is summarized in Supplemental Figure S1.

### Cell counting assay via hemocytometer

Preparation of cells for counting was achieved via established protocols^18,31^ and visualized on a Zeiss PrimoVert benchtop microscope (Dublin, CA, USA). Unless otherwise stated, cell counts were done on trypsinized, single-cell suspensions with a hemocytometer and the mean of the four cell-count measurements was calculated and rounded to the nearest integer.

### Bioluminescence imaging

For all bioluminescence work, we used genetically-modified GBM2, GBM39 and U87-MG whereby the glioblastoma cells were transfected with lentiviral vectors that expressed either firefly luciferase (fLuc for GBM39) or a fusion protein of GFP and firefly luciferase (GFP/fLuc for GBM2 and eGFP-fLuc for U87-MG) or a Renilla luciferase -Red Fluorescence protein fusion (RLuc-RL8 for KR158B)^32,33^. Cells were transduced using viral supernatants, and expression of luciferases was confirmed by measuring cellular luciferase activity (IVIS Spectrum; Perkin Elmer, Waltman, MA) in the presence of d-Luciferin (0.3 mg/mL final concentration) for fLuc and coelenterazine (1 μg/mL) for rLuc.

### Scanning electron microscopy (SEM)

5,000 (low seeding condition) to 50,000 (high seeding condition) U87-MG/eGFP-fLuc cells or PCS-201 fibroblast cells were deposited onto 13 mm glass coverslips and then prepared for TTFields experiments under a protocol described in Supplemental Figure S1. Cells were grown under standard tissue culture incubator conditions (37 ^o^C, 95% O_2_, 5% CO_2_). At the end of the TTFields-exposed and TTFields-unexposed experiments (1 day for high-seeding conditions and 3 days for low-seeding conditions), the coverslips were processed for SEM. Full details of SEM methodology are in legends of Supplemental Figure S10 and S16. All ROI analyses were performed in a blinded manner in which neither the individual responsible for SEM image acquisition nor the one performing data analyses knew of the experimental conditions for the samples. A third individual had possession of the sample identities.

### Chemical reagents

Unless otherwise stated, all chemicals were purchased from Selleckchem Inc. (Houston, TX, USA), Thermo-Fisher Scientific (Waltham, MA, USA), or Sigma-Aldrich (St. Louis, MO, USA). Purified firefly luciferin or firefly luciferase (SRE0045-2MG) as well as the Ethidium D apoptosis kit (11835246001) were purchased from Sigma Aldrich Inc (St. Louis, MO). Dextran-FITC of molecular weights 4, 20, and 50 kDa (FD4, FD20 and FD50), were purchased from Sigma Aldrich Inc. as well. 5-aminolevulinic acid (5-ALA, AAA16942ME) and the AnnexinV-APC kit (50712549) were purchased from Thermo-Fisher Scientific Inc (Waltham, MA). Supplemental Table S1 summarizes the reagents used in this study.

### Statistical analysis

The PRISM 7.0 software (GraphPad Software Inc., La Jolla, CA, USA) was used to determine whether the data were normally distributed. Normally distributed data were analyzed with two-way Student’s *t*-test or analysis of variance (ANOVA) comparisons of means, while non-normally-distributed data were analyzed with non-parametric analyses (e.g., Mann–Whitney *U* test comparison of medians). The level of statistical significance was set at alpha = 0.05. Bonferroni or Dunnet post-hoc corrections were employed to adjust alpha for multiple comparisons. All data are presented as range, mean ± standard deviation, median [interquartile range], or percent. In all figures, the levels of statistically significant differences are represented by: *p < 0.05, **p < 0.01, and ***p < 0.001.

## Results

### Induction of TTFields increases BLI in luciferase-expressing glioblastomas

**Fig. 2:**
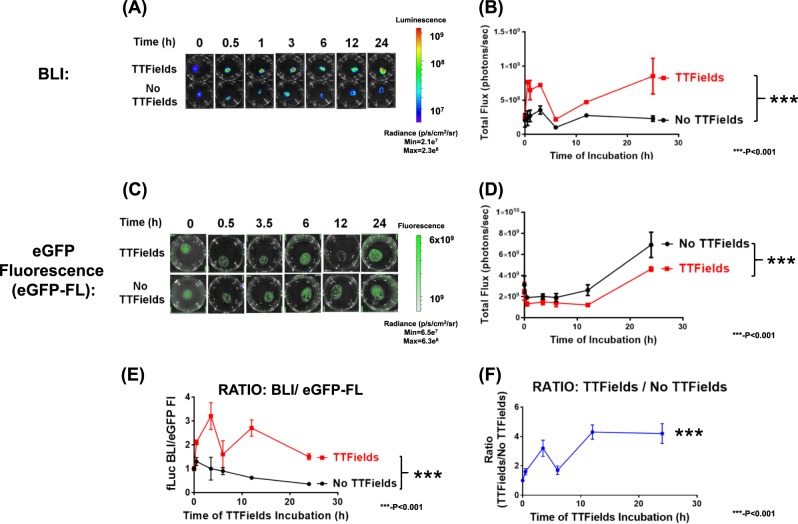
Increased bioluminescence signal in U87-MG/eGFP-fLuc cells exposed to TTFields as shown by: **a** Representative panel of bioluminescent imaging (BLI) scans as a function of time in TTFields vs. no TTFields conditions. **b** Temporal quantification of BLI data in **a**. Significant difference (***p < 0.001) in plot between no TTFields and TTFields. Effect of TTFields on eGFP fluorescence in U87-MG/eGFP-fLuc cells as shown by **c** time course of representative panels for TTFields-exposed vs. TTFields-unexposed U87-MG/eGFP-fLuc and **d** temporal quantification of fluorescence data in **c**. Significant difference (***p < 0.001) in plot between no TTFields and TTFields. **e** Effect of TTFields on the fLuc bioluminescence (fLuc-BLI) over eGFP fluorescence (eGFP-FL) ratio for U87-MG/eGFP-fLuc cells as a function of length of TTFields exposure and **f** effect of TTFields exposure vs. non-exposure on the fLuc-BLI/eGFP-FL ratio as a function of TTFields exposure time (hours). For both **e** and **f**, there is a significant difference in ratio (***p < 0.001) between TTFields exposed and TTFields non-exposed. Two-way ANOVA analysis and *n* = 3 experiments per data point in each panel (**b**, **d**, **e** and **f**)

TTFields (4 V/cm, 200 kHz, 0.5–24 h duration) significantly increased bioluminescence intensity (BLI) of U87-MG/eGFP-fLuc cells compared to unexposed conditions (Fig. 2a). This increase in BLI occurred as early as 30 minutes after commencement of TTFields and continued to 24 h of TTFields exposure (Fig. 2a). When ROI quantification was performed, the time course of BLI intensity for the TTFields-exposed samples was significantly elevated compared to TTFields-unexposed samples (Fig. 2b, *p* < 0.0001, two-way ANOVA, TTFields vs. no TTFields). The presence of TTFields did not significantly increase eGFP fluorescence (eGFP-FL) over the course of the experiments. When ratios of BLI over eGFP-FL was compared between TTFields vs. no TTFields samples, there was a significantly augmented ratio with respect to time of TTFields incubation for the TTFields samples (Fig. 2e, f, *p* < 0.0001, two-way ANOVA, TTFields vs. no TTFields). TTFields significantly decreased activity of purified firefly luciferase (Supplemental Figure S2, *p* < 0.01, two-way ANOVA, TTFields vs. no TTFields).

Application of TTFields over time on another patient-derived glioblastoma cell line, GBM2/GFP-fLuc also induced a time-dependent increase in bioluminescence in TTFields-exposed GBM2/GFP-fLuc cells (Supplemental Figure S3A, B, *p* < 0.0001, two-way ANOVA, TTFields vs. no TTFields). This same effect by was observed in a murine astrocytoma cell line (KR158B) that was genetically modified to express Renilla luciferase-red fluorescent protein fusion protein (Supplemental Figure S3C-D, *p* < 0.0001, two-way ANOVA, TTFields vs. no TTFields). Renilla luciferase activity is not dependent upon ATP and magnesium.

### Effect of TTFields on uptake of membrane-associating reagents

**Fig. 3:**
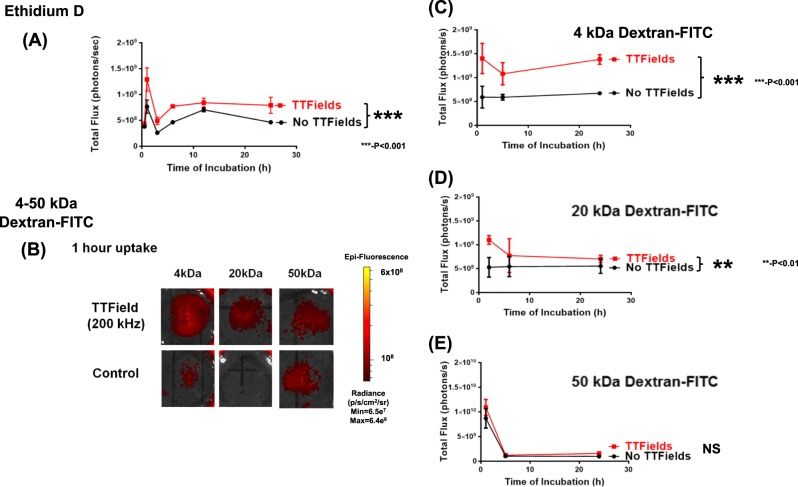
**a** Increased uptake of Ethidium D in U87-MG cells treated with TTFields, compared to the no TTFields condition (***p < 0.001). The effect of TTFields on the binding and uptake of Dextran-FITC of varying molecular weights in U87-MG cells. **b** A representative panel of Dextran-FITC fluorescence at 1 h incubation for 4, 20, and 50 kDa Dextran-FITC. Fluorescence scale bar shown on right. Impact of TTFields on the time course of Dextran-FITC uptake, **c** 4 kDa Dextran-FITC (***p < 0.001), **d** 20 kDa Dextran-FITC (**p < 0.01), and **e** 50 kDa Dextran-FITC (*p* = 0.26, not significant), compared to that of no TTFields. All statistical comparisons were based upon 2-way ANOVA analyses with each data point represented by *n* = 3 experiments. APC, allophycocyanin, Ethidium D, ethidium bromide, FITC, fluorescein isothiocyanate

Under our studied conditions, TTFields did not induce any significant degree of apoptosis in the U87-MG cells (Supplemental Figure S4). However, ethidium D uptake was significantly increased when the U87-MG/eGFP-fLuc cells were subjected to 200 kHz TTFields (Fig. 3a, *p* < 0.0001, two-way ANOVA, TTFields vs. no TTFields). Ethidium D permeates through both the plasma membrane and the nuclear membrane and intercalates into genomic DNA^34^. Thus, these findings suggest that TTFields can have an effect on the permeability of plasma membranes in U87-MG/eGFP-fLuc cells.

Dextran-FITC is known to bind and intercalate into the plasma membrane^35–37^. When U87-MG cells were subjected to 1 h of 200 kHz TTFields, there was a significant uptake of Dextran-FITC of molecular weights 4 kDa and 20 kDa, compared to no TTFields exposure, but there was no significant difference in uptake for 50 kDa Dextran-FITC (Fig. 3b–e). Over a timeframe of 0.5–24 h exposure, we found a significant increase in the uptake of 4 kDa Dextran-FITC compared to TTFields-unexposed samples (Fig. 3c, *p* < 0.0001, two-way ANOVA, TTFields vs. no TTFields), a significant increase in uptake of 20 kDa Dextran-FITC under TTFields exposure (Fig. 3d, *p* < 0.01, TTFields vs. no TTFields) and no significant difference in uptake of 50 kDa Dextran-FITC under TTFields exposure (Fig. 3e not significant, TTFields vs. no TTFields).

### Effect of TTFields on 5-aminolevulinic (5-ALA) acid uptake: single U87-MG culture

**Fig. 4:**
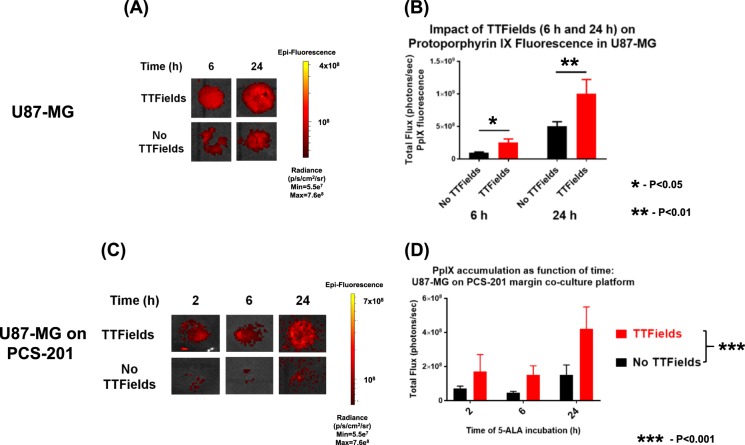
Effect of TTFields on 5-aminolevulinic acid (5-ALA) uptake as shown. **a** representative protoporphyrin IX (PpIX) fluorescence panel for TTFields-unexposed vs. TTFields-exposed U87-MG cells after 6 and 24 h of exposure. Scale bar on right used for both 6 h and 12 h post-exposure data. **b** Quantitation of images in **a** showed significant increase in PpIX signals in TTFields exposed cells compared to no TTFields, at both 6 h (*p* = 0.047) and 24 h (*p* = 0.01) time points. All monovariant statistical comparisons between no TTFields vs. TTFields samples done by Student’s *t*-test for *n* = 3 experiments per time point. **c**, **d** Effect of TTFields on U87-MG glioblastoma cells co-cultured with PCS-201 fibroblast cells. **c** Representative fluorescent panels of 5-ALA uptake (and subsequent PpIX fluorescence, Ex = 558 nm, Em = 583 nm) for no TTFields (top row) vs. TTFields (bottom row) conditions. Duration of exposures are 2, 6, and 24 h. **d** Quantification of time course of PpIX accumulation (and thus accumulation of fluorescent flux as expressed as photons/s) in the glioblastoma-fibroblast co-culture platform under TTFields exposed vs. unexposed conditions (*p* < 0.001). Statistical analyses consisted of two-way ANOVA for no TTFields vs. TTFields conditions, and *n* = 3 experiments per time point. Schematic of co-culture platform is shown in Supplemental Figure S4

We investigated the effects of TTFields on uptake of 5-ALA in glioblastoma cells. Because it is difficult to distinguish the margin between tumor and normal cells using the present 5-ALA bioassay^20,22^, we hypothesized that measurement of PpIX fluorescence would address this issue. We investigated whether permeation of 5-ALA through the cellular membrane and into the glioblastoma cells could be increased with TTFields exposure. U87-MG cells were exposed or unexposed to TTFields, each for durations of 6–24 h. TTFields exposure resulted in significantly increased uptake of 5-ALA into U87-MG/eGFP-fLuc cells as early as 6 h of TTFields exposure (Fig. 4a, b, *p* = 0.047, Student’s *t*-test, TTFields vs. no TTFields) and this increase was maintained with prolonged TTFields exposure of 24 h (Fig. 4a, b, *p* = 0.011).

### Effect of TTFields on 5-aminolevulinic acid uptake: U87-MG GBM on PCS-201 fibroblast co-cultures

To distinguish differences in 5-ALA uptake between glioblastoma and normal cells, a co-culture was developed where U87-MG cells were seeded in the center of a bed of PCS-201 fibroblasts and (Supplemental Figure S5) were subjected to TTFields or to no TTFields. Fluorescent and brightfield photomicrographs confirmed the presence of discrete glioblastoma (red arrows) vs. fibroblast (white arrows) cell regions in the co-culture set-up (Supplemental Figures S6–S7). When co-cultures were stained with hematoxylin and eosin (H&E), photomicrographs (Supplemental Figure S6) revealed reduced numbers of GBM cells (purple/dark pink stains) infiltrating into the fibroblast periphery (light pink) for TTFields-exposed samples. Without TTFields exposure, the GBM cells formed many pockets of adherent neurospheres (Supplemental Figure S6, dark spots on 1× images) as was previously reported^18,38^. Fluorescence images showed increased PpIX fluorescence in glioblastoma vs. fibroblast cells in the co-culture platforms (Supplemental Figure S7) that were subjected to TTFields for 6 h. PpIX fluorescence accumulated over time but the rate of fluorescence intensity increase was significantly augmented (Fig. 4c, d, *p* < 0.001, two-way ANOVA, TTFields vs. no TTFields) for TTFields-exposed co-cultures compared to TTFields-unexposed co-cultures. In a separate set of experiments, by 24 h of TTFields application, the ratio of PpIX fluorescence intensity in the U87-MG glioblastoma cells over the surrounding PCS-201 fibroblast cells was significantly increased (Supplemental Figure S8, *p* = 0.043, two-way ANOVA, TTFields vs. no TTFields).

### SEM shows that TTFields alters membrane morphology of U87-MG/eGFP-fLuc cells

**Fig. 5:**
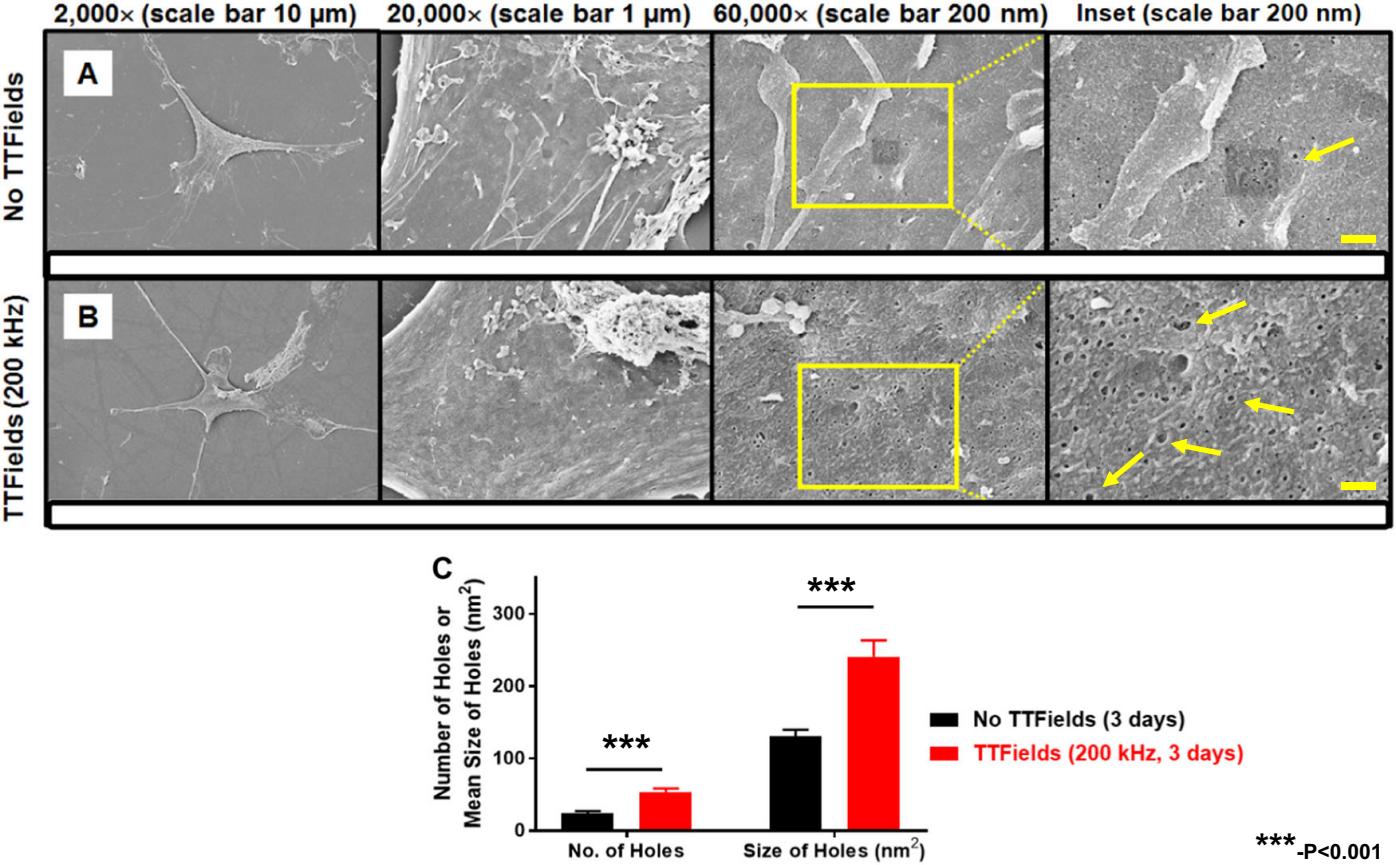
Scanning electron micrograph (SEM) comparison of plasma membrane holes in glioblastoma cells unexposed or exposed to TTFields. **a** Representative SEM images of a U87-MG/eGFP-fLuc cell unexposed to TTFields for 3 days with sparse holes in the plasma membrane. **b** Representative SEM images of a U87-MG/eGFP-fLuc cell exposed to TTFields for 3 days demonstrate more holes and of larger size in the plasma membrane, compared to that of cells not exposed to TTFields (Wilcoxon rank-sum analysis). **c** Quantification and comparison between TTFields unexposed and exposed cells of the number and size of holes with area ≥ 51.8 nm^2^ (equivalent to a 4-nm radius circle, or 9 pixels^2^ on the 60,000× magnification images) within a 500 nm-radius circular region of interest. The minimum hole size cut-off was based on the 3.3 and 5.0 nm Stokes radii of 20 kDa and 50 kDa Dextran-FITCs, respectively. From left to right, magnification levels in **a** and **b** are 2,000× (black scale bar 10 µm), 20,000× (black scale bar 1 µm), and 60,000× (black scale bar 200 nm) and final panel column on the extreme right, where yellow scale bar represents 200 nm scale. Yellow arrows point to representative holes on cellular membranes. Coverslips from three experiments per condition were used, and at least 5 cells per coverslip were analyzed for hole count and size, in a double-blind manner. Qualitative comparison of changes to the plasma membrane in cells seeded at higher density for 24 h of TTFields exposure vs. no TTFields exposure is shown in Supplemental Figure S9

**Fig. 6:**
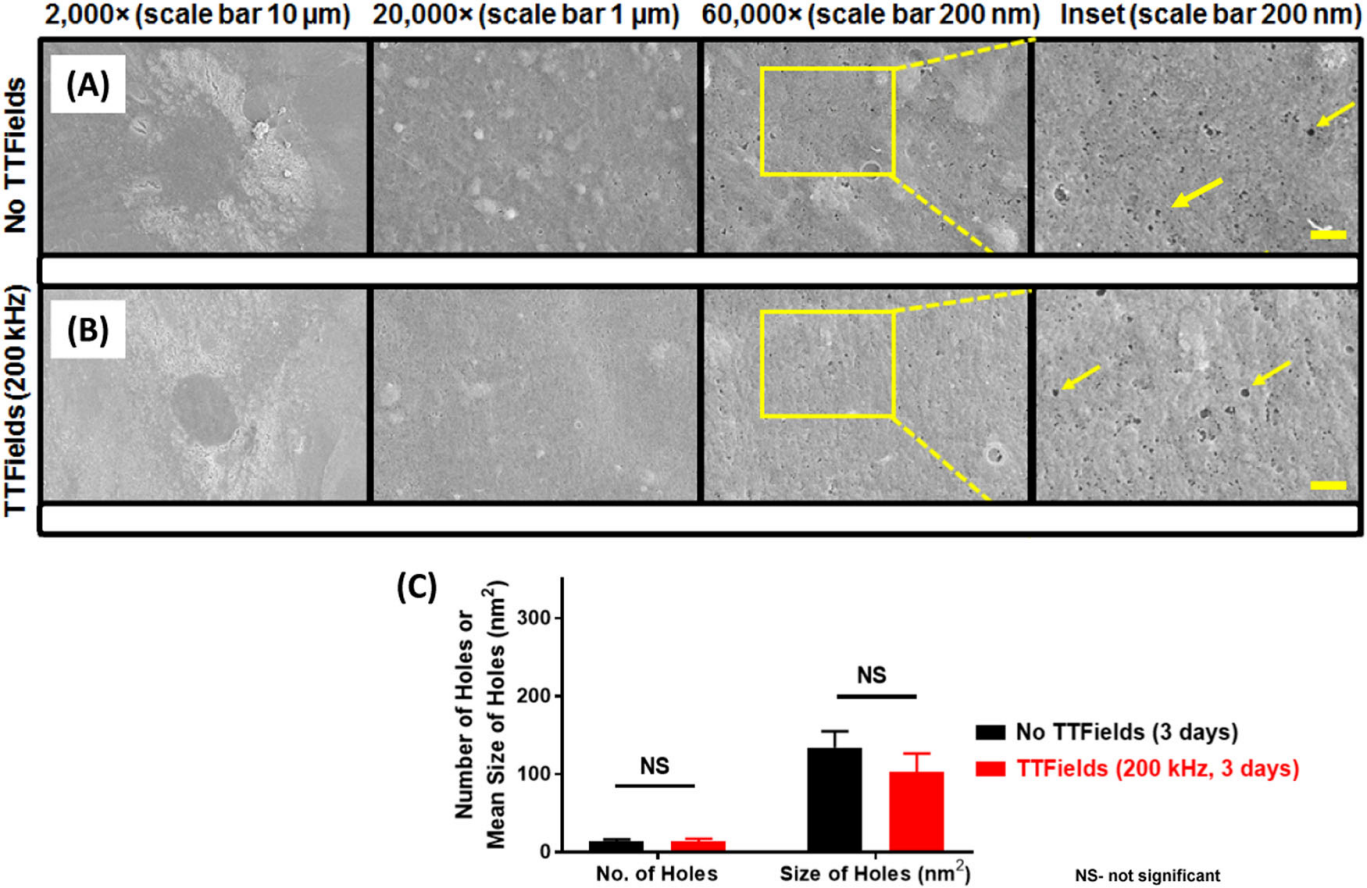
Scanning electron micrographs (SEM) of normal human PCS-201 cells seeded at low density (5,000 cells per 13 mm glass coverslip, see Supplemental Figure S1). The cells were grown under standard tissue culture conditions (37 ^o^C, 95% O_2_, 5% CO_2_). Non-TTFields-exposed cells (**a**) were left under those conditions for the duration of the study. Other cells (**b**) were exposed to TTFields for 72 h. **c** Quantification and comparison between TTFields unexposed and exposed cells of the number and size of holes with area ≥ 51.8 nm^2^ (equivalent to a 4-nm radius circle, or 9 pixels^2^ on the 60,000× magnification images) within a 500 nm-radius circular region of interest. The minimum hole size cut-off was based on the 3.3 nm and 5.0 nm Stokes radii of 20 kDa and 50 kDa Dextran-FITCs, respectively. There was no significant difference in the number or size of holes between the TTFields unexposed and exposed normal human PCS-201 cells (Wilcoxon rank-sum analysis). From left to right, magnification levels in **a** and **b** are 2,000× (black scale bar 10 µm), 20,000× (black scale bar 1 µm), and 60,000× (black scale bar 200 nm) and final panel column on the extreme right where yellow scale bar represents 200 nm scale. Yellow arrows point to representative holes on cellular membranes. Coverslips from three experiments per condition were used, and at least 5 cells per coverslip were analyzed for hole count and size, in a double-blind manner

Figure 5a, b shows representative SEM images of low-density (5,000 cells/coverslip) U87-MG/eGFP-fLuc cells that were either not exposed to TTFields (Fig. 5a) or exposed to TTFields for 3 days (Fig. 5b). There was a significantly increased number of holes greater than 51.8 nm^2^ in size (equivalent to 9 pixels^2^ on 60,000× magnification) within the ROI of TTFields-exposed cells (53.5 ± 19.1) compared to the TTFields-unexposed cells (23.9 ± 11.0), (*p* = 0.0002, univariate Mann–Whitney test). Average size of the holes within the ROI was also significantly greater in TTFields-exposed cells (240.6 ± 91.7 nm^2^) compared to TTFields-unexposed cells (129.8 ± 31.9 nm^2^), (Fig. 5c, *p* = 0.0005 (univariate Mann–Whitney test)). In contrast to U87-MG cells, TTFields did not significantly alter the size nor the number of holes in normal human fibroblast cells (Fig. 6).

The effects of a 24-h exposure to TTFields on the plasma membranes of U87-MG cells seeded at high density are shown in Supplemental Figure S10. Topological alterations of the membrane surfaces are best seen with subpanels with the 2–4 µm scale bars. For no TTFields samples, the cell surface appeared to be covered in densely matted, elongated and flattened membrane extensions, similar to membrane ruffles and contiguous with the cellular membrane. In contrast, after 24 h of exposure to TTFields, the densely matted and elongated structures were replaced by short, bulbous and bleb-like structures. TTFields did not appear to alter the membrane morphology of normal human PCS-201 cells (data not shown).

### The effect of TTFields on membrane permeability is reversible

**Fig. 7:**
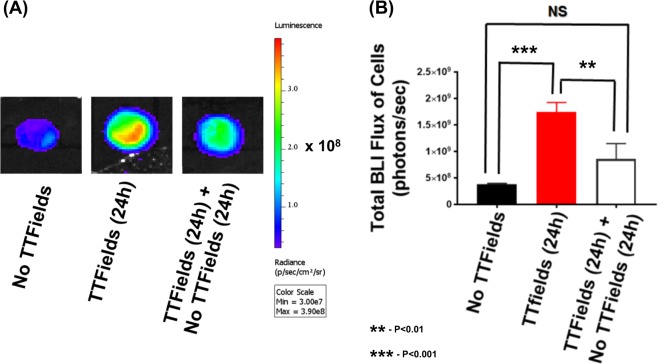
Study showing the reversibility of TTFields’ effects on bioluminescence activity of U87-MG/eGFP-fLuc cells. Cells were subjected to the conditions of: (1) standard, control tissue culture settings of 37^o^C, 95% O_2_, 5% CO_2_ and no exposure to TTFields, (2) 24 h of TTFields exposure, and (3) 24 h of TTFields exposure followed by additional 24 h of no TTFields exposure. All experimental conditions were done in triplicate and statistical analysis calculated via 2-way ANOVA. NS stands for not significant. **a** Representative panel of bioluminescent imaging (BLI) scans as a function of the three aforementioned conditions (i.e., no TTFields, TTFields (24 h), TTFields (24 h) followed by no TTFields (24 h)) and **b** quantification of BLI data in **a**. BLI, bioluminescent imaging, eGFP, enhanced green fluorescence protein, fLuc, firefly luciferase

To assess the reversibility of the effect of TTFields on cancer cells, U87-MG/eGFP-fLuc cells were subjected to three conditions: (1) No TTFields exposure, standard cell culture conditions (37 ^o^C, 95% O_2_, 5 %CO_2_), (2) TTFields exposure (24 h) and (3) TTFields exposure (24 h) followed by no TTFields exposure (24 h). The readouts of BLI, PpIX fluorescence and Dextran-FITC fluorescence were acquired (Fig. 7 and Supplemental Figures S11-S15). The presence of TTFields (24 h) significantly increased BLI flux compared to no TTFields exposure (Fig. 7, *p* < 0.0005, two-way ANOVA, TTFields vs. no TTFields, Supplemental Figures S11) but this increase was significantly attenuated when the cells were re-introduced to the no TTFields condition for 24 h (Fig. 7, two-way ANOVA, *p* < 0.005, TTFields [24 h] vs. TTFields [24 h] followed by no TTFields [24 h]). A similar pattern of reversible readouts occurred with PpIX fluorescence (Supplemental Figure S12A, *p* < 0.0005, two-way ANOVA, TTFields vs. no TTFields and *p* < 0.0004, TTFields vs. TTFields followed by no TTFields) and for 4 kDa Dextran-FITC fluorescence (Figure 12B, *p* < 0.05, two-way ANOVA, TTFields vs. no TTFields; and *p* < 0.05, TTFields vs. TTFields followed by no TTFields). For each experimental set, eGFP fluorescence did not significantly change (Supplemental Figures S11 and S13). SEM investigations also revealed that the significant augmentation in both the number of holes (Supplemental Figure S16-S17, *p* = 0.007, two-way ANOVA, TTFields vs. No TTFields) and the size of holes (Supplemental Figure S17, *p* = 0.0007, two-way ANOVA, TTFields vs. No TTFields) by TTFields were reversible as well, after 24-h of no exposure.

## Discussion

Previous studies have focused on the effects of TTFields on the nucleus (e.g., microtubules^39^), septin^14^, mitochondria, and autophagy^16^. To our knowledge, this is the first study to report the effects of TTFields on cancer cellular membrane integrity. We confirmed the phenomenon of increased cellular membrane permeability for glioblastomas in the presence of TTFields across multiple human GBM cell lines. The readout employed to validate the hypothesis included bioluminescence imaging (Figs. 2, 7 and Supplemental Figures S2/S11), fluorescence imaging (Figs. 2, 3, 4 and Supplemental Figures S4/S7/S8/S13/S15), and scanning electron microscopy (Figs. 5, 6 and Supplemental Figures S10/S16/S17). Studies of TTFields in combination with chemotherapies have shown both therapeutic additivity^6,40,41^ and synergy^18,42^. Future investigations should uncover why certain chemotherapies display additivity while other chemotherapeutics show synergy when combined with TTFields. For this study, we posited that TTFields mediates improved accessibility to cancer cells. Several experiments showed the reversibility of the TTFields effect on membranes thus demonstrating a causal relationship between TTFields and the increase in membrane permeability. Such observations also suggest that TTFields could be used to tune drug accessibility to cancer cells.

Our investigation into the cell permeability hypothesis of TTFields action was initiated partly because of our initial observation of increased bioluminescence in luciferase-expressing GBM cells by TTFields. We postulated that TTFields induced increased permeability in the cellular membranes of GBM cells. Increased GBM cell permeability to D-luciferin as measured by BLI was not due to the effects of TTFields on luciferase itself, but rather due to an increased influx of its substrate D-luciferin into the cells engineered to express the firefly luciferase. Furthermore, this finding held true for both ATP-dependent (FLuc) and ATP-independent luciferase (RLuc). Therefore, despite a preliminary report suggesting that intracellular ATP was increased in CT26 colorectal carcinoma cells exposed to TTFields^43^, the observation of increased glioblastoma cell membrane permeability in the setting of TTFields exposure suggests an independent phenomenon. An increased expression or activation of luciferase due to TTFields exposure could not have explained the increased BLI signal because in these cells the luciferase enzyme was controlled by the same promoter as was eGFP, and an increase in fluorescence signal was not observed in the same cells. However, exposure to TTFields may affect cellular metabolism that would be manifested by changes in ATP levels, alterations in membrane morphology and shifts in oxygen consumption.

Some key findings supporting the permeability hypothesis came from the Dextran-FITC validation experiments (Fig. 3b–e, Supplemental Figure S15). The accessibility of the cell membrane to small probes in the setting of TTFields was tested with FITC-labeled dextrans, which resulted in an increase in influx of 4 kDa (Stokes’ radius ~1.4 nm^44^) and 20 kDa (Stokes’ radius ~3.3 nm^44^) but not 50 kDa dextrans (Stokes’ radius ~5 nm^44^). This suggests that TTFields cause GBM cells to become more permeant to substances as large as 20 kDa, but no greater than 50 kDa. For reference (Supplemental Table S1), the luciferin and coelenterazine substrates are of small enough molecular weight to be accessible through the membrane with TTFields exposure. d-luciferin (substrate for Firefly luciferase) has a molecular weight of 280.3 g/mol (~280 Da)^45^, coelenterazine H (substrate for Renilla luciferase) has a molecular weight of 407.5 g/mol (~408 Da)^46^,5-ALA has a molecular weight of 167.6 g/mol (169 Da), consistent with the Dextran-FITC findings.

Our SEM findings are reminiscent to those reported by Bouakaz^47^. We showed that at low seeding density, 3 days of TTFields exposure caused a significant increase in the number and size of holes greater than 51.8 nm^2^ in area, compared to the no TTFields condition (Fig. 5). This hole size cut-off represents a circle of radius 4.1 nm, which is the Stokes’ radius of a FITC-dextran molecule with a size of 20–40 kDa (Supplemental Table S1). Thus, the difference in cell membrane disruption visualized by SEM confirms the indirect observations from our FITC-dextran studies. Interestingly, exposure of normal human fibroblasts (PCS-201) to TTFields caused no significant increase in the number or size of cellular membrane holes, thus suggesting that the permeability effect may have some specificity to cancer cells. Qualitatively, for U87-MG cells, there was a clear onset of bulbous, bleb-like structures due to a 24-h exposure to TTFields under high seeding density (Supplemental Figure S10). The appearance of these structures is consistent with increased permeability in the outer membrane^48^ and the induction of apoptosis^49–51^ although in our hands, there is little evidence of an apoptotic phenotype with a 24-h TTFields exposure. In our studies, high-density PCS-201 cells displayed no such changes with TTFields exposure (data not shown) thus suggesting again, the specificity of the TTFields effect for cancer cells.

Although we did not synchronize the cell cycle for our experiments, the doubling time of the U87-MG cells is ~48 h and given that, TTFields exert their maximal antiproliferative effect on dividing cells, this could explain the lack of observed abundant apoptosis after a 24-h TTFields exposure. An alternative interpretation may lie in reports that cellular blebbing may confer resistance to cellular lysis^52^. A previous report in unsynchronized glioblastoma cells demonstrated that 72 h of TTFields exposure induced cell death with a marked proportion of Annexin V-positive cells^16^. Using transmission electron microscopy, they also showed signs of autophagy including autophagosomes, swollen mitochondria, and a dilated endoplasmatic reticulum^16^. In contrast, we used SEM to better visualize the effects of TTFields specifically on the plasma cell membrane.

The increase in membrane permeability by TTFields may have clinical implications. Using the co-culture platform of human GBM cells layered on top of normal human fibroblast cells, we studied the impact of TTFields on the uptake of 5-aminolevulinic acid (5-ALA) into GBM cells.^53^ We showed that TTFields exposure resulted in significantly increased 5-ALA uptake in the GBM cells compared to the fibroblast cells^53^. In June 2017, 5-ALA was approved by the Food and Drug Administration for clinical use in the United States to assist neurosurgeons in delineating the tumor-normal brain border during glioma resection^54^. Future clinical studies may consider pre-treating glioma patients with TTFields prior to 5-ALA administration, possibly to enhance the delineation of the infiltrative tumor margin during tumor resection. In addition, the impact of TTFields on blood-brain permeability may warrant investigation.

With regard to detecting and measuring the effects of TTFields on cancer cells, the majority of cell culture-based studies to date have focused on cell count/viability as the primary readout^8,14,16,18,55^. This is based on the prevailing understanding that TTFields interferes with mitosis of rapidly dividing tumor cells, which results in cancer cell death. In addition, computational modeling studies of TTFields in cell culture are currently driven by cell count as the primary outcome of the model^11,56,57^. As additional mechanisms of action of TTFields (e.g., increase in cellular permeability described in the current study) emerge, additional read-outs based on these mechanisms will follow suit.

Recurrence of GBM is inevitable and the median time to first recurrence despite standard therapy is approximately 7 months^58,59^. In clinical applications of TTFields to patients with GBM, the data suggest that increased compliance and duration of TTFields use correlates with improved survival^60–62^. TTFields compliance (≥75% vs. <75%) was an independent predictor of overall survival in the retrospective analysis of the full EF-14 trial dataset^2^ and the duration of use of TTFields was also found to affect overall survival^60^. Taken together, these data may serve as clinical correlates of the observed effects in the cell cultured-based TTFields experimental setting. Namely, we observed a correlation between the length of TTFields exposure and the duration of its effect on cell membrane permeability after cessation of TTFields. At lengths of TTFields exposure of 0.5–3 h, the duration in BLI augmentation (compared to no TTFields conditions) lasted about 5 min. However, at TTFields exposures of 12–25 h, this difference in BLI between TTFields and no TTFields conditions lasted for more than 20 min (Supplemental Figure S9A). Likewise, a re-analysis of the data reported by Ram et al.^60^ shows that the percent increase in overall survival (in patients treated with TTFields plus temozolomide vs. temozolomide alone) jumped from 32% after 1 year of TTFields exposure to 551% after 5 years of TTFields exposure, respectively (Supplemental Figure S9B).

This study should be considered in the context of its limitations. We cannot confirm our results in an animal model of glioblastoma because a practical device that delivers TTFields to rodent brain does not yet exist. In addition, we focused our work in glioblastoma cells using the 200 kHz TTFields frequency, because currently FDA approval exists only for glioblastoma and only at this frequency. The novelty of the findings is the first report of a direct effect of TTFields on increasing, in a reversible manner, plasma membrane permeability in glioblastoma cells, which has clinical implications as described above. Nevertheless, we propose that our studies will influence future treatments of glioblastomas. Given the increasing interest in TTFields within the scientific and clinical literature, the future foreshadows additional insights into mechanisms of TTFields.

## Electronic supplementary material

Supplementary Material
