# Supplementary material for: Tumor treating fields increases membrane permeability in glioblastoma cells
Source: Cell Death Discov. 2018 Dec 5;4:113. doi: 10.1038/s41420-018-0130-x (PMC6281619; doi:10.1038/s41420-018-0130-x)
Supplement: Supplementary file 1 — Supplementary Material [file 41420_2018_130_MOESM1_ESM.docx]

**List of Supplemental Tables and Figures**

**Supplemental Table S1**

| **Reagent** | **Structure** | **Molecular Weight*** | **Radius (nm)** | **Reference** |
| --- | --- | --- | --- | --- |
| 5-Aminolevulinic acid | 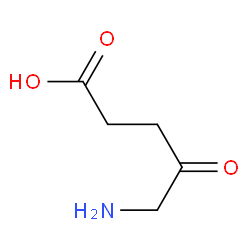[1] | 131.1 g/mol [1] | 0.18±0.005 (based on molar volume of 106.5±3.0 cm^3^ [1]) | (1) |
| Coelenterazine H | 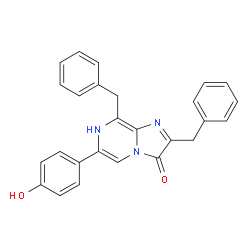[2] | 407.5 g/mol [2] | 0.54±0.01 (based on molar volume of 323.0±7.0 cm^3^ [2]) | (2) |
| Coelenterazine | 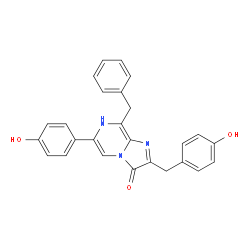[3] | 423.5 g/mol [3] | 0.54±0.01 (based on molar volume of 323.0±7.0 cm^3^ [3]) | (3) |
| Fluorescein isothiocyanate (FITC)-dextran (4 kDa) | 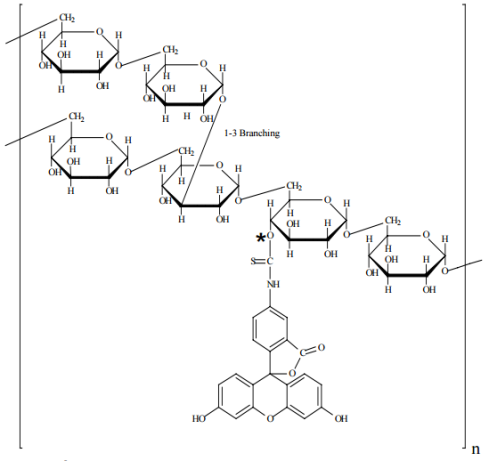* denotes the site of attachment of FITC is assumed to be randomly associated with any free hydroxyl group [4] | 4,000 g/mol [4] | 1.4 (Stokes radius) [4] | (4) |
| Fluorescein isothiocyanate (FITC)-dextran (20 kDa) |  | 20,000 g/mol [4] | 3.3 (Stokes radius) [4] |  |
| Fluorescein isothiocyanate (FITC)-dextran (50 kDa) |  | 50,000 g/mol [4] | 5 (Stokes radius) [4] |  |
| D-Luciferin | 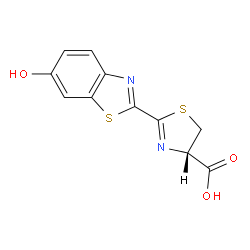[5] | 280.3 g/mol [5] | 0.26±0.01 (based on molar volume of 154.5±7.0 cm^3^ [5]) | (5) |
| Renilla Luciferase | 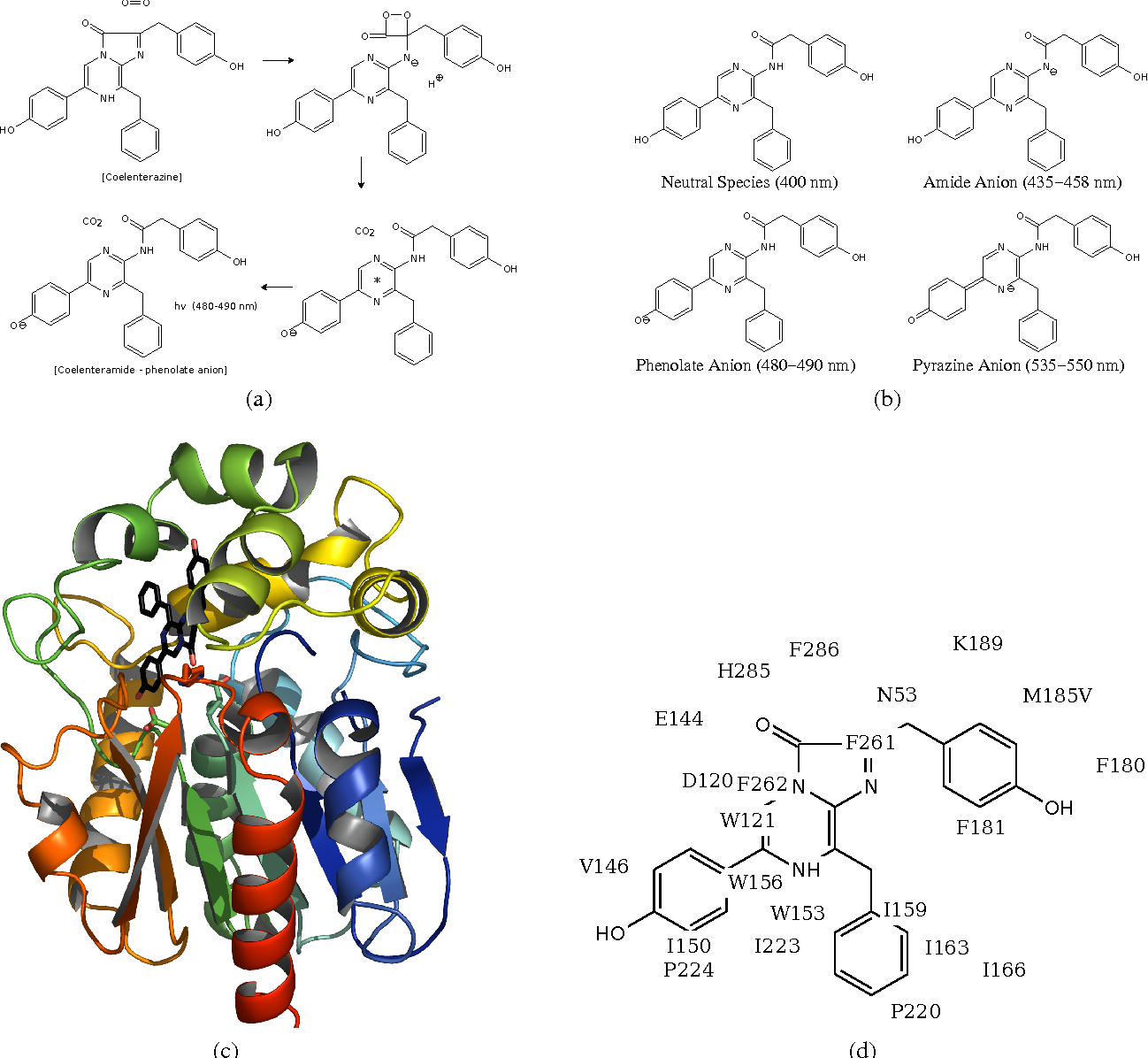 [6] | 36 kDa [6] | 25.5-27 (Stokes radius) [7,8] | (6-8) |
| PpIX (Protoporphyrin IX) | 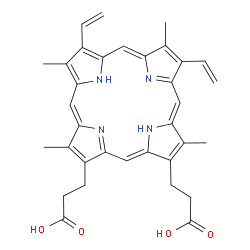[9] | 562.7 g/mol [9] | 0.56±0.11 (based on molar volume of 433.2±3.0 cm^3^ [9])  or  101.8 (based on aggregates with mean size 4.42×10^-6^ nm^3^ in a pH 5.0 solution [10]) | (9, 10) |
| Firefly Luciferase | 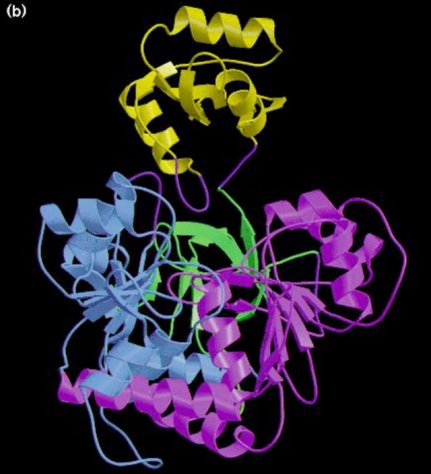 [12] | 62 KDa [11] | 2.54±0.03 (radius of gyration) [12] | (11, 12) |
| Ethidium-D (Ethidium Bromide) | 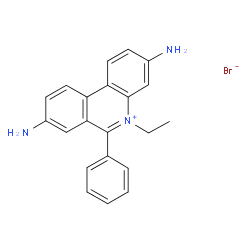 [13] | 394.3 g/mol [13] | Not found | (13) |
| Annexin V, allophycocyanin conjugate (APC Annexin V) | 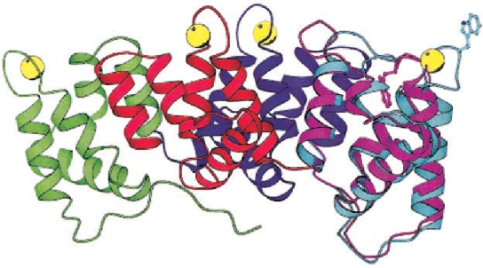[15] | ~36 kDa [14] | Not found | (14, 15) |
| Green Fluorescent Protein (GFP) | 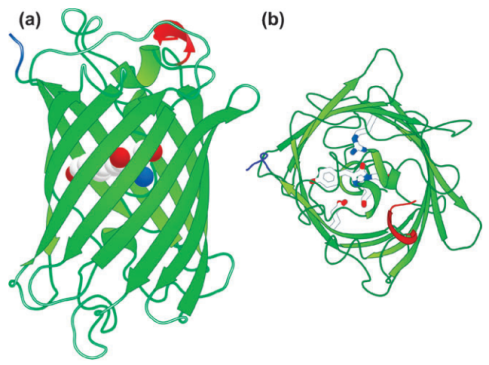 [17] | 27 kDa [16] | 2.82 nm (Stokes radius) [16] | (16, 17) |

D-luciferin (substrate for Firefly luciferase) has a molecular weight of 280.3 g/mol (~280 Da) (18), coelenterazine H (substrate for Renilla luciferase) has a molecular weight of 407.5 g/mol (~408 Da) (19),5-ALA has a molecular weight of 167.6 g/mole (169 Da), consistent with the Dextran-FITC findings.


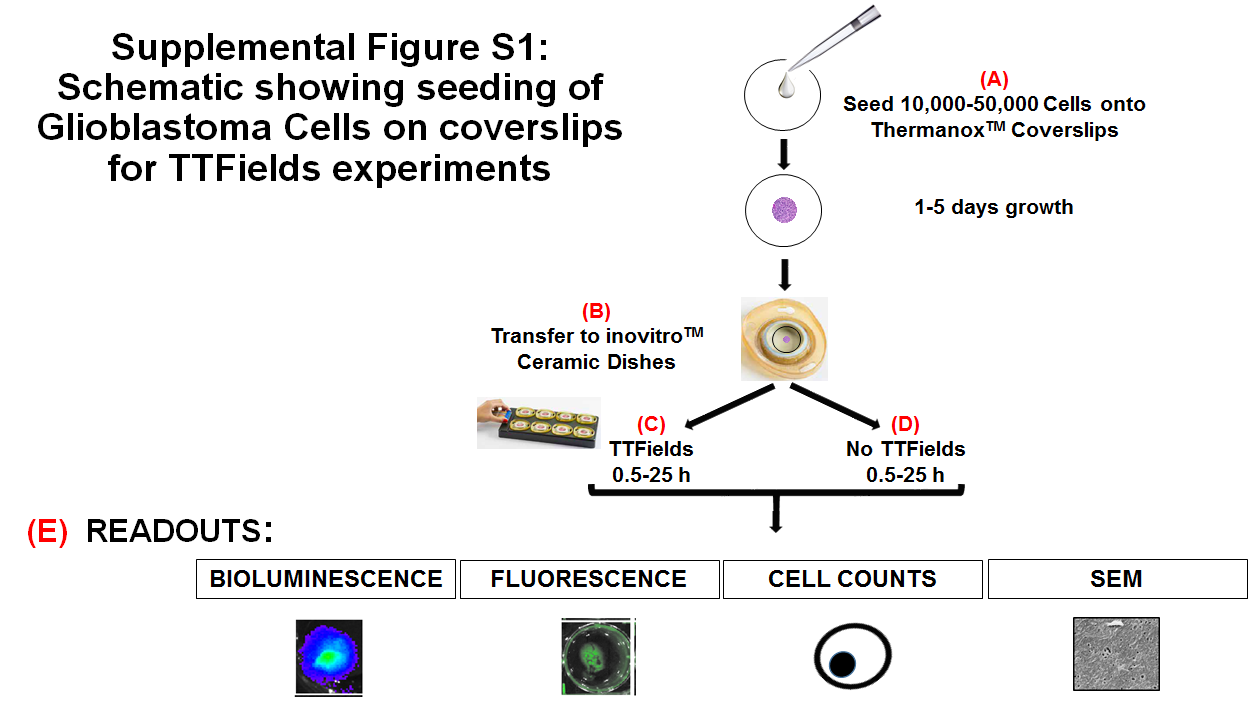


**Supplemental Figure S1:** Schematic of a standard TTFields experiment. (A) The seeding of 10,000-50,000 single glioblastoma cells in the middle of a 22-mm diameter coverslip. Cells remained on the coverslips for 1-5 days in order to achieve growth phase, then transferred to (B) the wells of an inovitro^TM^ TTFields system ceramic dish (Novocure, Ltd., Haifa, Israel). The ceramic dishes were placed either in (C) base plates that are connected to the power box of the inovitro^TM^ TTFields system and then into a special incubator or (D) into a conventional tissue culture incubator (37^o^C, 95% air, 5% CO_2_ for both incubators) for the no TTFields samples. Cells exposed or unexposed to TTFields for 0.5-25 hours. (E) Afterwards, the samples were processed for bioluminescence imaging, fluorescence imaging, cell counting or SEM analysis. For reversibility experiments, cells were exposed to TTFields for 24 hours and then transferred to no TTFields conditions for an additional 24 hours.

**Supplemental Figure S2: Effect of TTFields on Activity of Purified fLuc**

**Bioluminescence:**

**Purified fLuc**

**(A)**


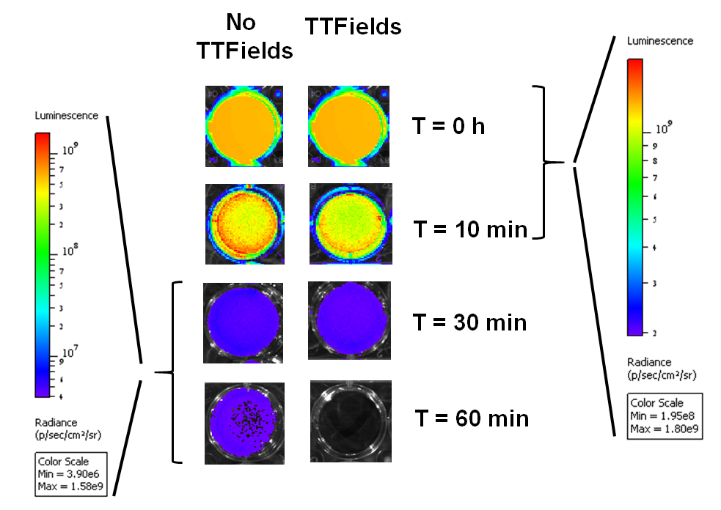


**10^9^**

**2x10^8^**

**(B)**

******

****-P<0.01**


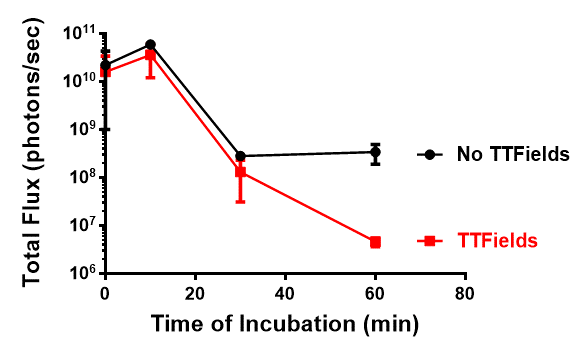


**Supplemental Figure S2:** Effect of TTFields on purified firefly Luciferase (fLuc) activity as displayed by (A) panel of BLIs of purified fLuc (no TTFields vs. TTFields) and (B) temporal quantification of BLI data in (C). Significant difference (**, p<0.01) in plot between no TTFields and TTFields. 2-way ANOVA analysis and n=3 experiments per data point in each panel (B). The elevation in bioluminescence was not due to a direct effect of TTFields on firefly luciferase activity because exposure of purified firefly luciferase to TTFields led to over a 1000-fold loss in enzymatic activity 60 minutes after initiation of TTFields.

**Supplemental Figure S3: Effect of TTFields on Activity of pcGBM2/GFP-fLuc and KR158B/GFP-fLuc**


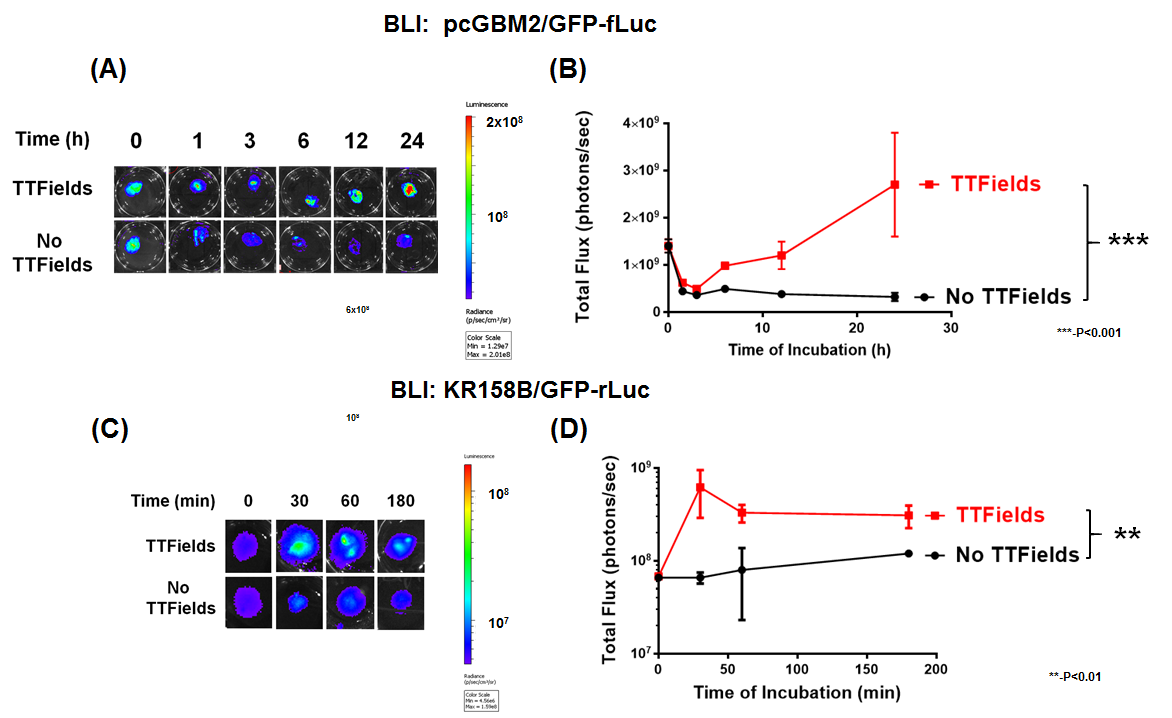


**Supplemental Figure S3:** Increase in bioluminescence signals in GBM2/GFP-fLuc cells treated with TTFields as shown by: (A) representative panel of bioluminescent imaging (BLI) scans as a function of time from start of no TTFields vs. TTFields conditions, and (B) temporal quantification of BLI data in (A). Statistically significant difference (***, p<0.001) in plot between no TTFields and TTFields. Effect of TTFields on bioluminescence signals of murine KR158B/rLuc-GFP cells as shown by: (C) representative panel of BLI as a function of time under no TTFields vs. TTFields conditions and (D) temporal quantification of BLI data in (C) Note y-axis is in log scale. Statistically significant difference (**, p<0.01) in plot between no TTFields and TTFields. 2-way ANOVA analysis and n=3 experiments per data point in each panel (B and D). fLuc = Firefly luciferase, rLuc = Renilla luciferase, GFP= green fluorescence protein.

**Supplemental Figure S4: Effect of TTFields on Apoptosis Index as assessed by Annexin V-APC Fluorescence**

**Annexin V-APC**


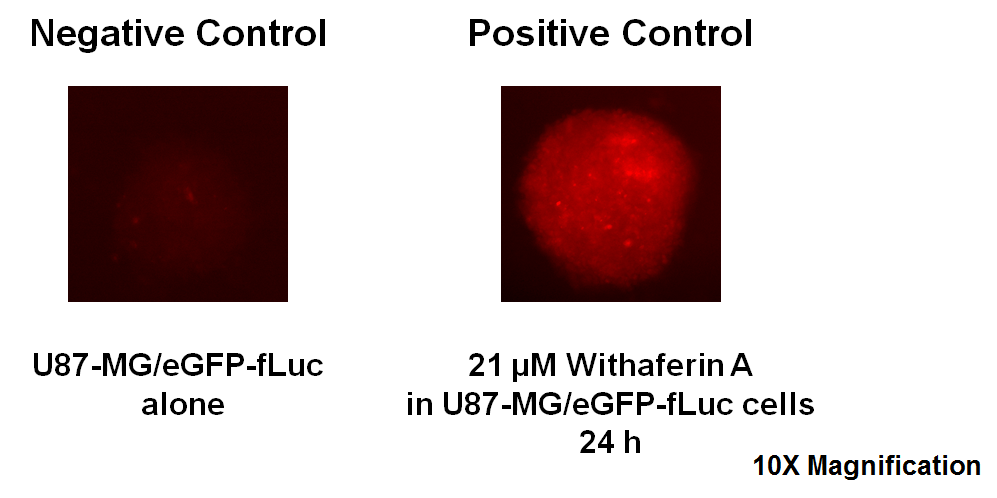


**(A)**

**(B)**


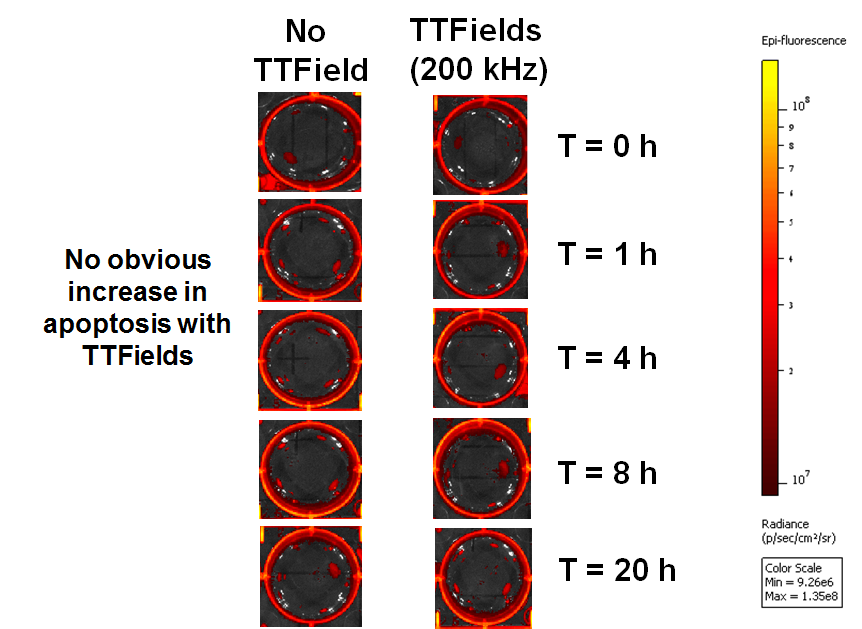


**10^8^**

**10^7^**

**Supplemental Figure S4**: Impact of tumor treating fields (TTFields) on uptake of Annexin V-APC in U87-MG cells. (A) Representative negative (0 µM Withaferin A, no TTFields) and positive controls (21 µM Withaferin A, no TTFields) for Annexin V-APC fluorescence in U87-MG cells and (B) representative panel of Annexin V-APC fluorescence as a function of time for no TTFields vs. TTFields conditions. There was no detectable uptake of Annexin V-APC for either no TTFields or TTFields conditions.

To test if the imposition of TTFields does affect membrane properties and thus membrane permeability, we explored the effect of TTFields on the behavior of fluorescently tagged reagents that bind to the cellular membrane. Initially we looked at the impact of TTFields on the binding of Annexin-V-APC to the phosphatidylserine in the inner membrane of U87-MG cells. Annexin-V-APC binding is a signature of early apoptosis which is characterized by ruffling of the membrane (20). A positive control to induction of apoptosis (addition of 21 µM Withaferin A to U87-MG cells (21)) was used to assess the visibility of Annexin-V-APC binding to U87-MG cells, and showed that such binding could be visualized via fluorescence microscopy (**Supplemental Figure S3A**) over TTFields-unexposed samples. However, when TTFields was applied to U87-MG cells, there was no visualization of Annexin-V-APC binding at any time point of exposure to TTFields (**Supplemental Figure S3B**). This suggests that TTFields, in the period that it was applied, did not induce any significant degree of apoptosis to the U87-MG cells.


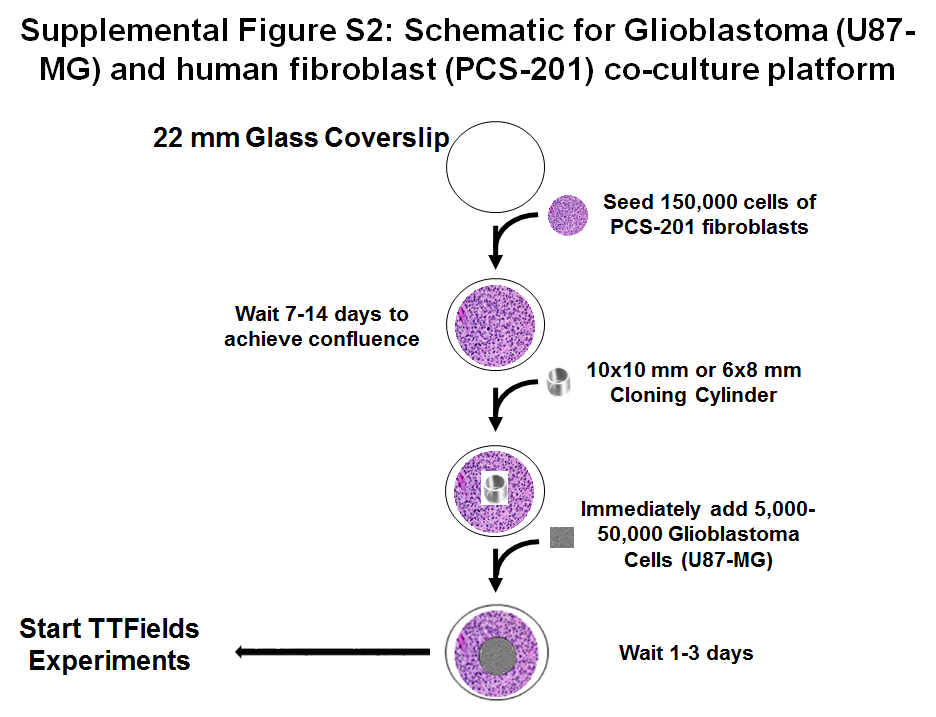


**Supplemental Figure S5: Schematic for Glioblastoma (U87-MG) and human fibroblast (PCS-201) margin co-culture**

**Supplemental Figure S5:** Schematic for set-up of U87-MG and PCS-201 co-culture platform prior to TTFields experiments. 150,000 PCS-201 fibroblast cells were seeded onto a 22 mm Thermanox glass coverslip and the cells allowed to incubate and to grow under standard tissue culture conditions (37^o^C, 95% air, 5% CO_2_) for 7-14 days. This allowed the fibroblasts to cover the whole slip. All coverslips housed in either a 6-well or a 12-well tissue culture plate. A 10×10 mm or a 6×8 mm cloning cylinder was then placed in the middle of the coverslip and 5,000-50,000 glioblastoma cells were seeded in the inside of the cylinder and allowed to settle overnight. The cells were then allowed to grow under standard tissue culture conditions for an additional 1-2 days. The coverslips were then processed for TTFields experiments.


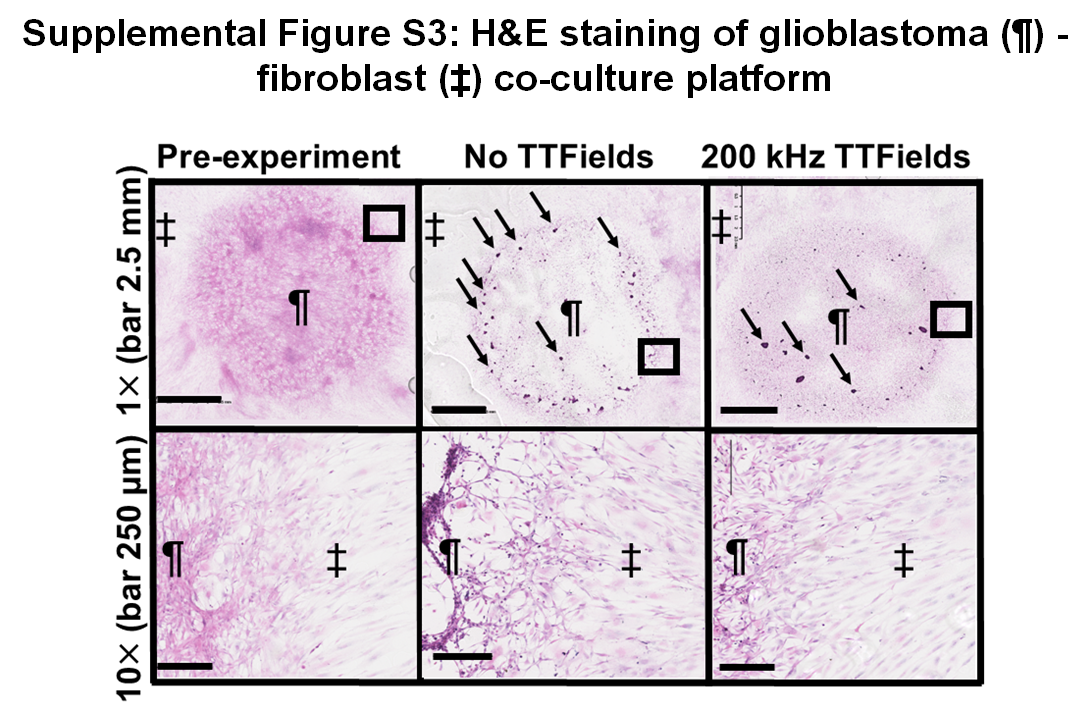

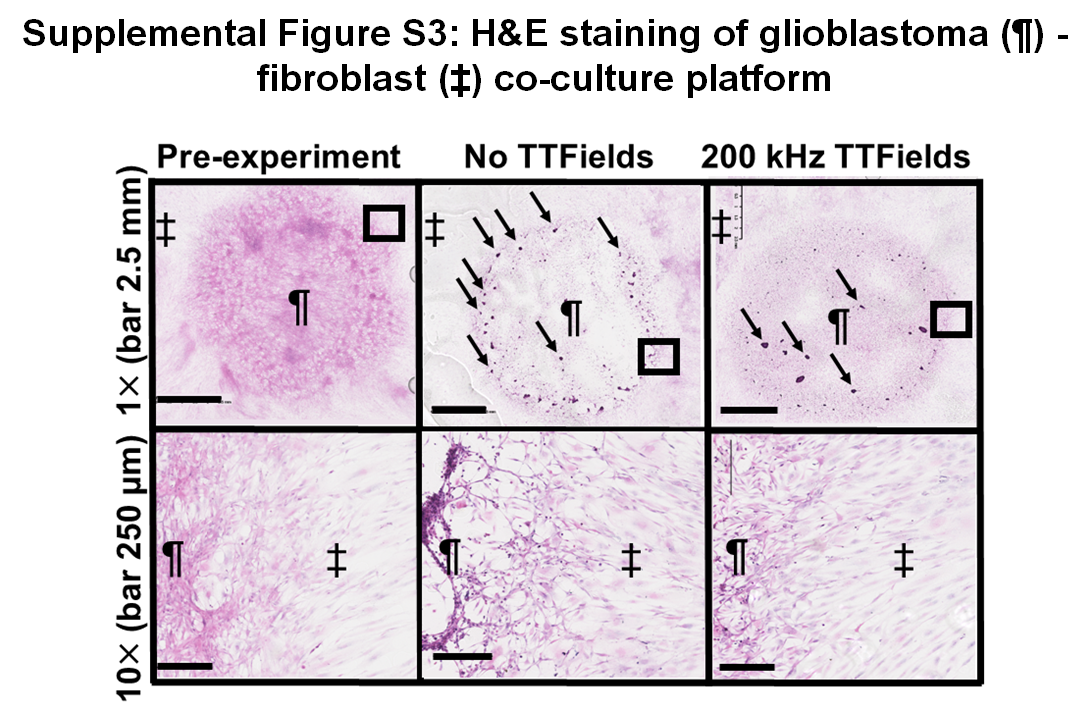


**Supplemental Figure S6: H&E staining of glioblastoma (¶) - fibroblast (‡) co-culture platform**

**Supplemental Figure S6:** Hematoxylin and eosin (H&E) staining of glioblastoma-fibroblast co-culture platform. H&E staining revealed U87-MG glioblastoma cells (¶, purple/dark pink) surrounded by normal human fibroblast PCS-201 cells (‡, light pink). The small boxes inside the 1× panels represent the region magnified at 10×, in which the U87-MG cells are shown on the left and PCS-201 on the right. The reduced number of GBM cells infiltrating into the fibroblast periphery due to TTFields is evident when compared to the no TTFields condition. In particular, without TTFields exposure, the glioblastoma cells formed many pockets of adherent neurospheres (black arrows), less of which are seen under exposure to TTFields. Black scale bars represent 2.5 mm (top row) and 250 µm (bottom row).


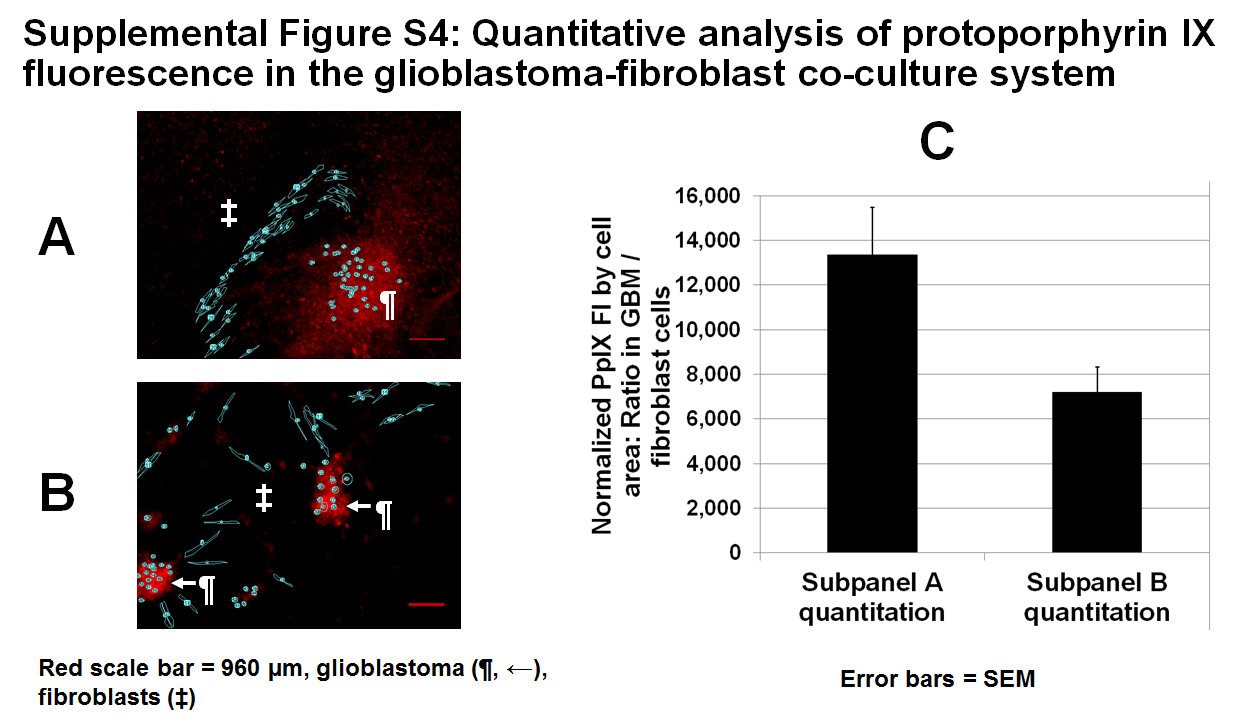


**Supplemental Figure S7: Quantitative analysis of protoporphyrin IX fluorescence in the glioblastoma-fibroblast co-culture system**

**Supplemental Figure S7:** Quantitative analysis of protoporphyrin IX fluorescence in the glioblastoma-fibroblast co-culture platform (**Supplemental Figure S5**). As described in the Methods section of the main manuscript, U87-MG glioblastoma (GBM) cells and PCS-201 fibroblast cells were co-cultured, 5-aminolevulinic acid (5-ALA, teal) was added, and protoporphyrin IX (PpIX) fluorescence microscopy (Cy5 intensity, red) was performed. Images were exported as bitmap files and analyzed in ImageJ version 1.38x (National Institutes of Health, Bethesda, MD). The images were converted to 8-bit gray scale and automated window/leveling was applied. Polygonal regions of interest (ROIs) were drawn around 25-40 GBM and fibroblast cells each at the GBM-fibroblast margin of the co-culture platform. The PpIX fluorescence intensity (FI) in each ROI was normalized to the cell area (FI/area, arbitrary units). (A) and (B) show the ROIs overlaid on the original fluorescence image (¶ and ← refer to the centrally located GBM cells and ‡ refers to the peripherally located fibroblast cells). (A) Shows the margin between a central mass of GBM cells surrounded by fibroblast cells as shown in **Figure 4C**. (B) Shows GBM neurospheres surrounded by fibroblast cells. (C) is a quantitative plot of the mean±SEM of the ratio of the FI/area in the GBM cells to the FI/area in the fibroblast cells, corresponding to subpanels (A) and (B). There was variability in the ratio of the FI/area measure in the GBM/fibroblast cells (13,356.7±2,111.9 in subpanel (A) and 7,194.1±1,137.5 in subpanel (B)), but both representative examples show the marked increase in PpIX fluorescence intensity in the GBM cells compared to the fibroblast cells, when normalized to cell area. This difference was accentuated at the cell population level upon exposure to TTFields as shown in **Supplemental Figure S8**.

**Supplemental Figure S8**


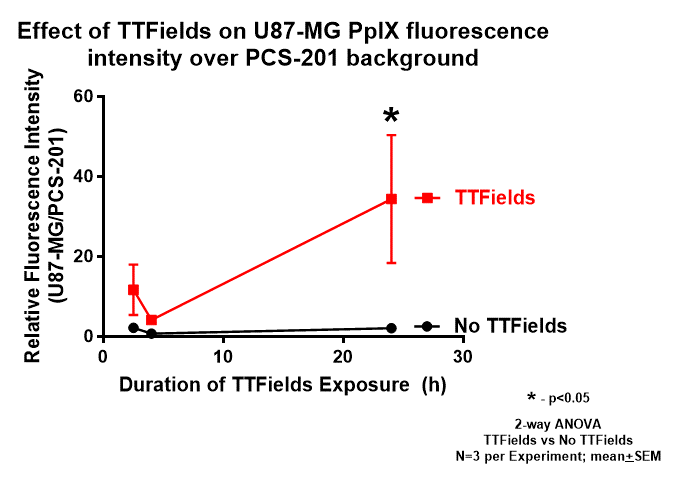


**Supplemental Figure S8:** Effect of TTFields on U87-MG protoporphyrin IX (PpIX) fluorescence intensity over PCS-201 background, in the co-culture platform. Living Image software version 4.5.4 (Perkin Elmer, Waltham, MA) was used for region of interest (ROI) analysis. The fluorescence intensity of the central glioblastoma ROI was divided by that of the peripheral fibroblast ROI to derive the relative fluorescence intensity (U87-MG / PCS-201), p=0.043 between no TTFields and TTFields conditions. Three independent experiments were performed per time point. 2-way ANOVA statistical analysis was performed using Prism version 7.04 (GraphPad, La Jolla, CA). The 5-ALA findings could have an impact on standard of care and treatment for glioblastoma patients. For example, the current clinical correlate to evaluating the effects of TTFields on cancer cell viability is based on reduction in glioblastoma size over serial magnetic resonance imaging (MRI) scans. A future clinical correlate to evaluating the effects of TTFields on the permeability of glioblastoma cell membranes could be clinical trials that evaluate the ability of TTFields to increase the intraoperative fluorescence signal delineating the tumor boundaries in glioblastoma patients who have been administered 5-ALA.

**Supplemental Figure S9: Relationship between length of TTFields exposure and survival rate**


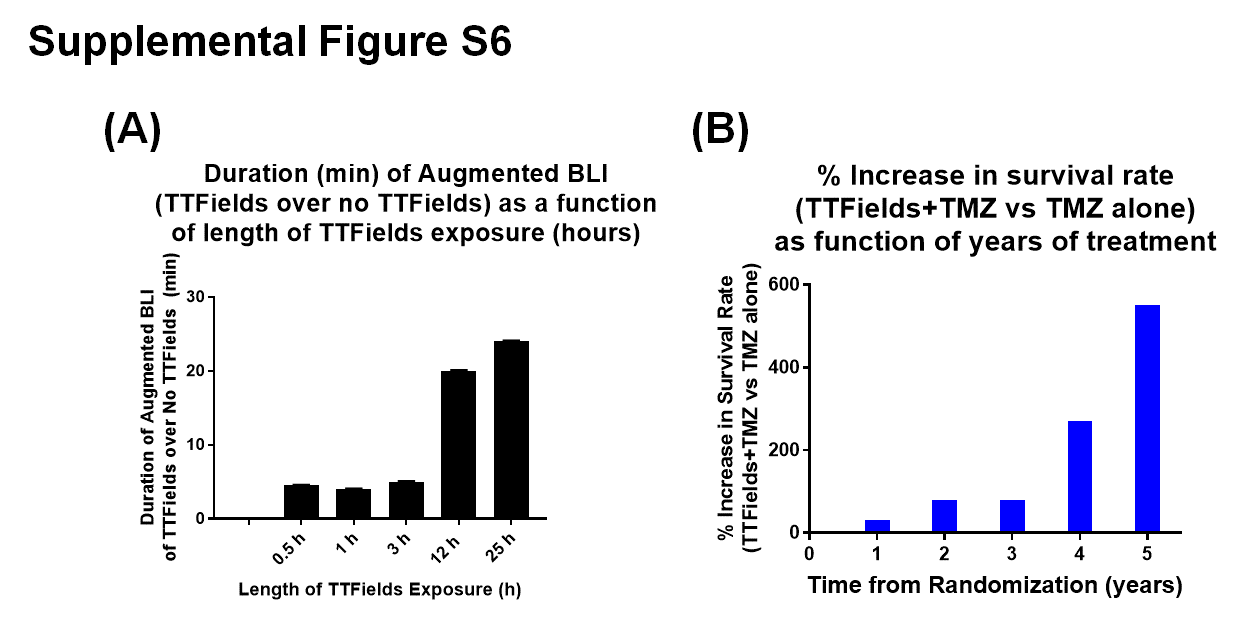


**Supplemental Figure S9:** Length of tumor treating fields (TTFields) exposure prolongs duration of bioluminescence (BLI) signal difference in glioblastoma cell culture and overall survival in glioblastoma patients. (A) Duration of BLI difference as a function of length of TTFields exposure for TTFields vs. no TTFields conditions. Living Image software version 4.5.4 (Perkin Elmer, Waltham, MA) was used to quantify the bioluminescence intensity of the U87/MG-eGFP-fLuc glioblastoma cells after fixed lengths of TTFields or no TTFields exposure (0.5, 1, 3, 12, and 25 hours). BLI images were acquired every 5 min after cessation of TTFields or no TTFields exposure. For each length of TTFields or no TTFields exposure, the post-exposure time when the BLI signal difference between the TTFields and no TTFields conditions became less than 10% was defined as the duration of BLI difference. This duration of BLI difference is plotted as a function of the length of TTFields or no TTFields exposure. This graph shows that after 3 hours of TTFields exposure, there was an increase in the duration of BLI difference between the TTFields and no TTFields conditions, indicating prolonged increased glioblastoma cell membrane permeability with longer duration of TTFields exposure. (B) Analogous patient findings with the clinical TTFields system showing that greater length of high compliance (>90%) leads to prolonged overall survival. Subpanel (B) is modified from the work of Ram Z, Kim CY, Nicholas GA, Toms S. (22) The percent increase in survival rate was calculated in the following manner for each year *i* post-randomization: [(A*_i_*-B*_i_*)/B*_i_*]×100%, where A*_i_* = overall survival rate in the TMZ+TTFields cohort and B*_i_* = overall survival in the TMZ only cohort, for each year *i* post-randomization.

**Supplemental Figure S10: Effect of TTFields on high density SEM for U87-MG/eGFP-fLuc cells**


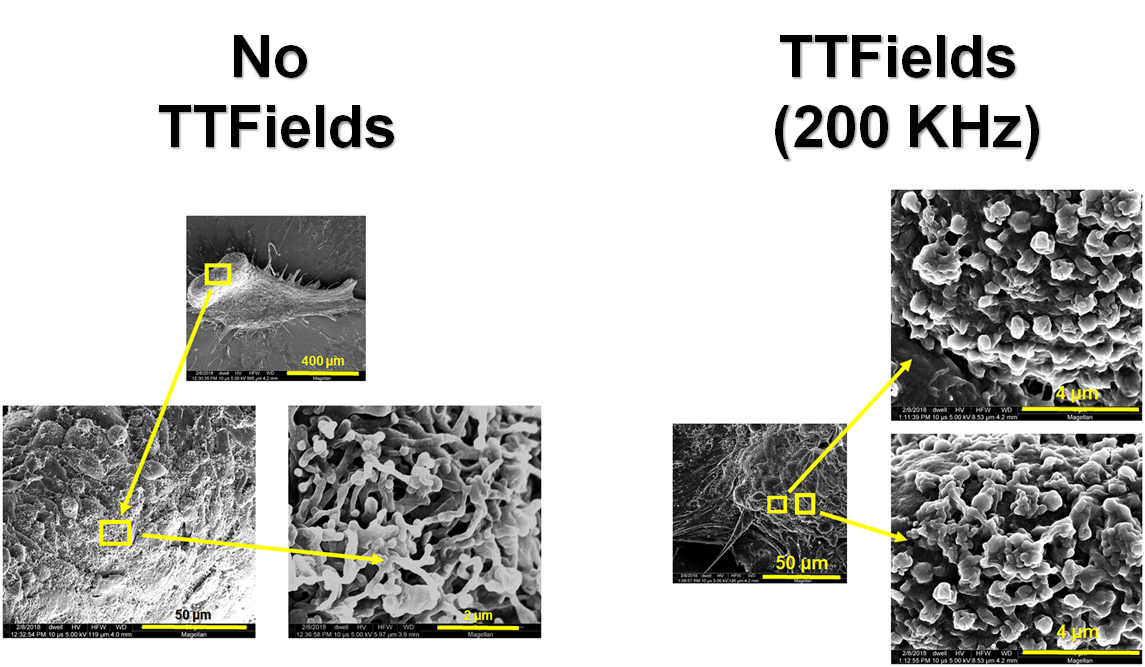


**Supplemental Figure S10:** Scanning electron micrographs of U87-MG/eGFP-fLuc cells seeded at high density (5µm to 400µm scale, red scale bars in each image) that were exposed (right panel) or not exposed (left panel) to TTFields for 24 or 72 hours. 5,000 (low seeding density, see **Figure 5**) or 50,000 cells (high seeding density, **current Figure**) were seeded onto 13 mm glass coverslips and then prepared for TTFields experiments under a protocol described in **Supplemental Figure S1**. Cells grown under standard tissue culture incubator conditions (37^o^C, 95% O_2_, 5% CO_2_). At the end of the TTFields-exposed or the TTFields-unexposed experiment (3 days for low seeding conditions and 24 hours for high seeding conditions), the coverslips were processed for scanning electron microscopy (SEM). Initially, the coverslips were fixed at room temperature with 4% paraformaldehyde/0.1M Na cacodylate buffer (pH 7.0) for 30 minutes. Then the solution was exchanged for 2% glutaraldehyde/0.1M Na cacodylate (pH 7.0) for another 30 minutes at room temperature. The fixative was then exchanged for 70% ethanol and stored on ice until the samples were submitted to either the Cell Sciences Imaging Facility (for the low seeding density conditions) or the Stanford Nano Shared Facility (for the high seeding density conditions).

Low seeding density conditions

Samples were critical point dried with liquid CO_2_ in a Tousimis Autosamdri-815B apparatus (Tousimis, Rockville, MD), mounted with conductive carbon tape onto 15 mm aluminum stubs (Electron Microscopy Sciences, Hatfield, PA), and sputter-coated with 50-100 Å of gold-palladium using a Denton DeskII Sputter Coater (Denton Vacuum, Moorestown, NJ). Visualization was performed with a Zeiss Sigma FE-SEM (Carl Zeiss Microscopy, Thornwood, NY) operated at 5 kV, using InLens and SE2 SE detection at a working distance of approximately 7 mm.

Visualization was achieved by SEM at 2,000×, 20,000×, or 60,000× magnification, and corresponding images over the cell bodies were acquired. TIFF images were captured at 2,048 × 1,536 pixels resolution and a line averaging noise reduction algorithm. 6-8 representative cells were imaged at each magnification level per coverslip (n=2-3/condition) by a staff member of the Stanford Cell Sciences Imaging Facility who was blinded to the experimental conditions.

For quantitation of the number and size of holes in the glioblastoma cell membrane at low seeding density under TTFields or no TTFields conditions, the high-magnification (60,000×) 8-bit images were imported into ImageJ version 1.38x (National Institutes of Health, Bethesda, MD). The level was set to 1 in order to enhance the visual contrast between the dark holes and the gray/white cell membrane. The image was then thresholded from 0 (lower limit) to 190-210 (upper limit), which filled in the holes (the darkest 1-3% of pixels within the image). A 500 nm-radius circular ROI was drawn over the cell body in an area containing smooth holes that were biological, as opposed to jagged-edged holes that represented processing artifacts (personal communication with Lydia-Marie Joubert, PhD, Director of Electron Microscopy Unit, Stellenbosch University, South Africa). Within the 500 nm-radius circular ROI, the number of cellular membrane holes with size ≥ 9 pixels^2^ (51.8 nm^2^, representing a circle of radius 4.1 nm) were counted and the average size of the holes within the ROI was calculated. The number and size of holes was then compared between the TTFields exposed and unexposed cells. Data acquisitions and quantifications were performed in a double blind manner. In other words, neither the technicians responsible for acquisition of the SEM images nor the individuals involved in ROI analysis were informed of the different experimental conditions of the samples. A third party was custodian of the sample key that delineated the experimental conditions. Such information was revealed once all analyses were completed.

High seeding density conditions

Images were acquired using a FEI Magellan 400 XHR Scanning Electron Microscope (Nanolab Technologies, Milpitas, CA). For the current Figure above, the effect of TTFields on glioblastoma cell membranes was most apparent in the subpanels at the 5 µm scale. For the no TTFields samples, the cell surface appeared to be covered with dense, matted and elongated structures (contiguous with the cellular membrane). After 24 hours of exposure to TTFields, the dense, matted structures disappeared, and they were replaced by short, bulbous structures.

**Supplemental Figure S11 (BLI reversibility panel)**

**BLI Media**


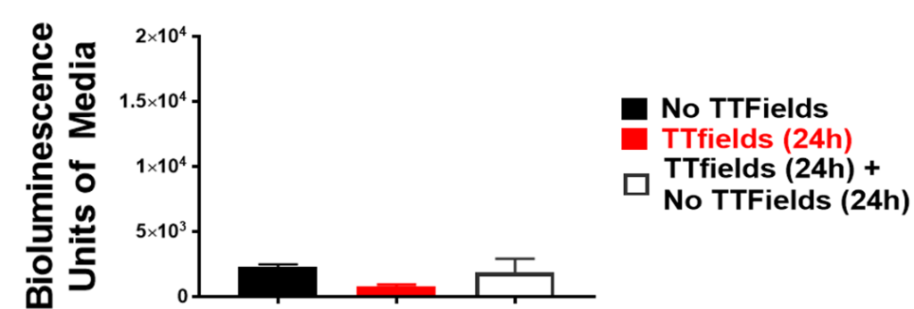


**(E)**


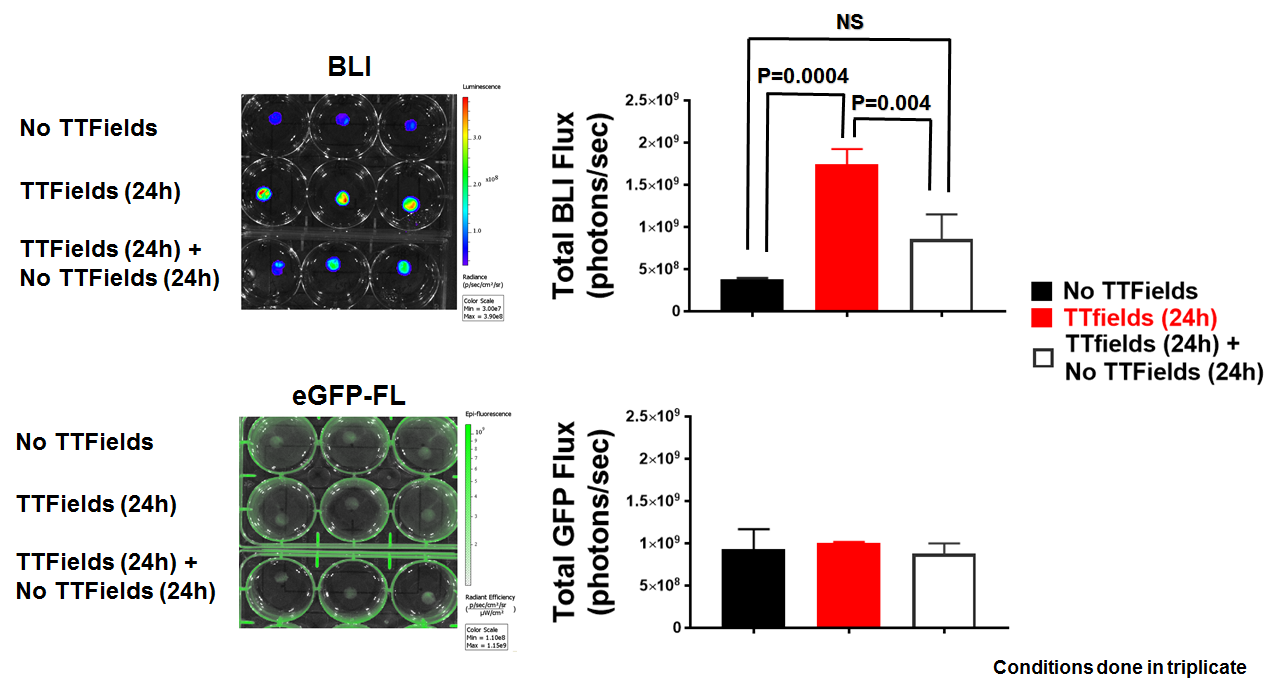


**(A)**

**(B)**

**(C)**

**(D)**

**eGFP-FL**

**BLI Cell**

***

**

_**_ **- p<0.01**

_***_ **- p<0.001**

**(C)**

**(D)**

**Supplemental Figure S11:** Effect of 24 h TTFields exposure (24h) on (A-B, E) firefly luciferase activity (from bioluminescence of oxidized D-luciferin) and on (C-D) eGFP fluorescence of U87-MG/eGFP-fLuc cells (A, B, C, D) and on (E) bioluminescence of the conditioned media from the U87-MG cell culture. Cells were seeded onto 22 mm glass coverslips, grown, and prepared for TTFields exposure according to the procedure described in **Supplemental Figure S1**. Cells were subjected to the conditions of: (1) standard, control tissue culture settings of 37^o^C, 95% O_2_, 5% CO_2_ and no exposure to TTFields, (2) 24 hours of TTFields exposure and (3) 24 hours of TTFields exposure followed by additional 24 hours of no TTFields exposure. All experimental conditions were done in triplicate and statistical analysis calculated via 2-way ANOVA. Bioluminescence imaging performed in the presence of 0.3mg/ml D-luciferin and recorded in an Ilumina II IVIS machine. eGFP fluorescence was collected by the same instrument with Ex= 465 nm and Em= 510 nm. Bioluminescence of media read by a Turner Biosystems 20/20 Luminometer. BLI – bioluminescent imaging, eGFP-FL-enhanced Green Fluorescence Protein fluorescence. Level of significance: ** represents p<0.01 and *** represents p<0.001.

**Supplemental Figure S12 (PpIX reversibility)**


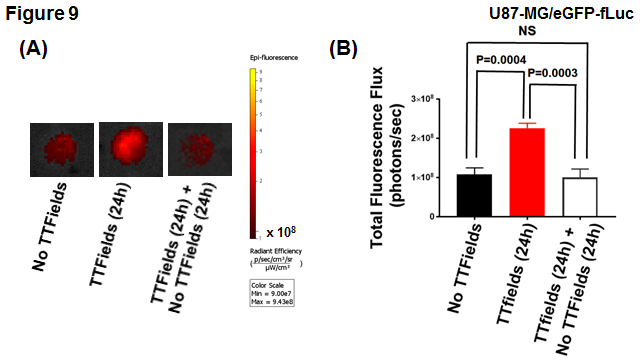


*******

*****’**

**_***_ - p<0.001**

**Supplemental Figure 12:** Study showing the reversibility of TTFields’ effects on 5-ALA uptake (as assessed by PpIX fluorescence) by U87-MG cells. Cells were subjected to the conditions of: (1) standard, control tissue culture settings of 37^o^C, 95% O_2_, 5% CO_2_ and no exposure to TTFields, (2) 24 hours of TTFields exposure and (3) 24 hours of TTFields exposure followed by additional 24 hours of no TTFields exposure. All experimental conditions were done in triplicate and statistical analysis calculated via 2-way ANOVA. NS represents no significant difference. (A) Representative panel of PpIX fluorescence imaging scans as a function of the three aforementioned conditions (i.e. no TTFields, TTFields (24 h), TTFields (24 h) followed by no TTFields (24 h)) and (B) quantification of PpIX fluorescence intensity in (A). 5-ALA = 5-aminolevulinic acid; PpIX = protoporphyrin IX; eGFP = enhanced green fluorescence protein; fLuc = firefly luciferase. *** represents level of significance of p<0.001.


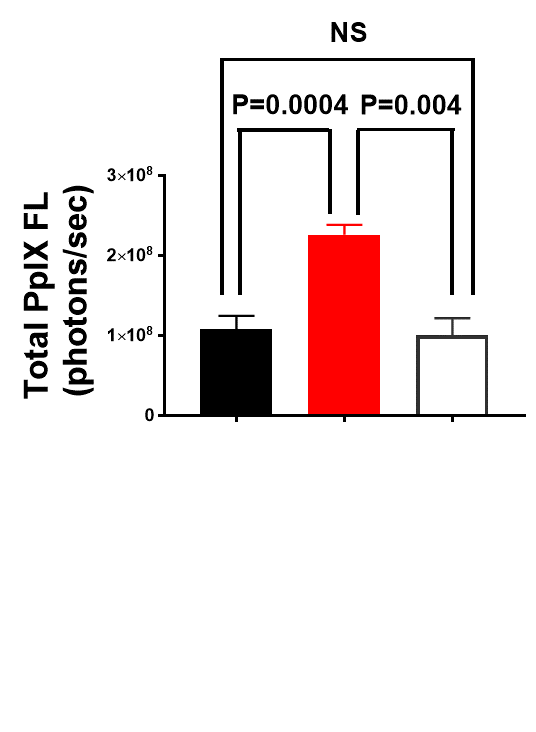
**Supplemental Figure S13 (PpIX reversibility panel)**

**(E)**
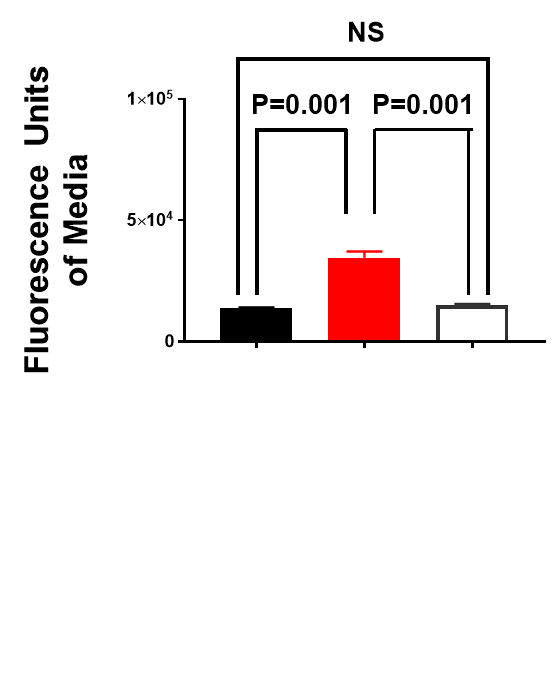


**PpIX-FL Media**


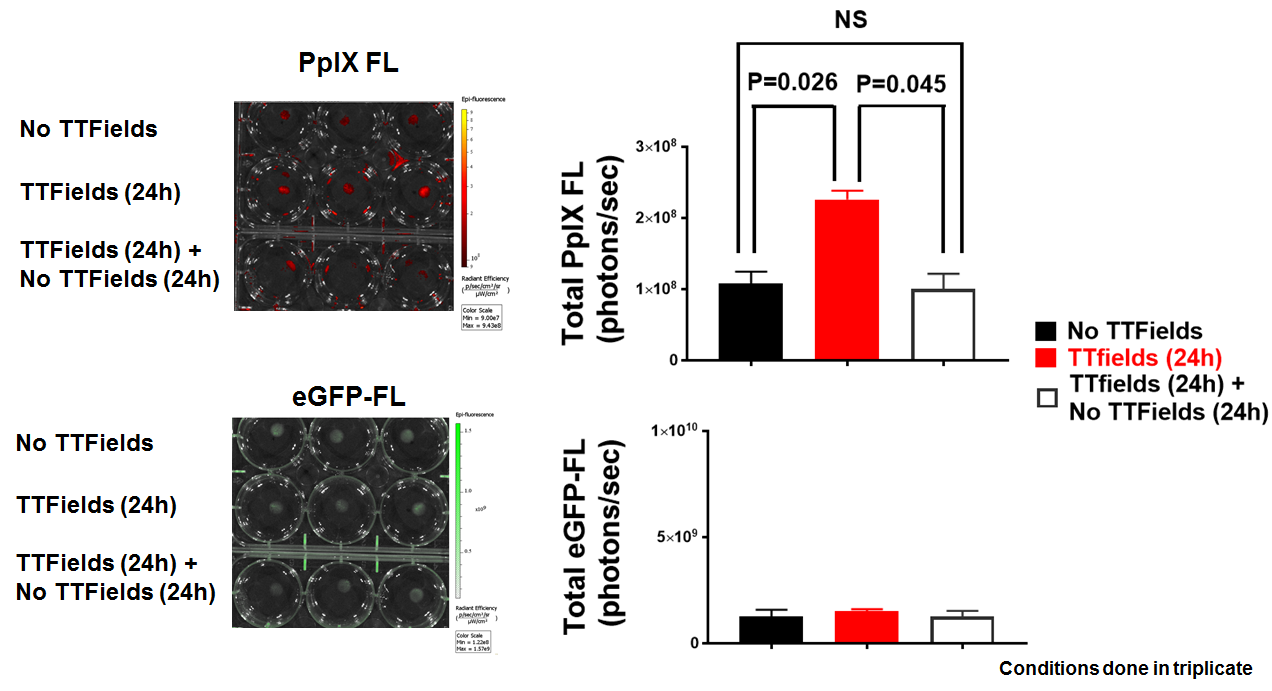

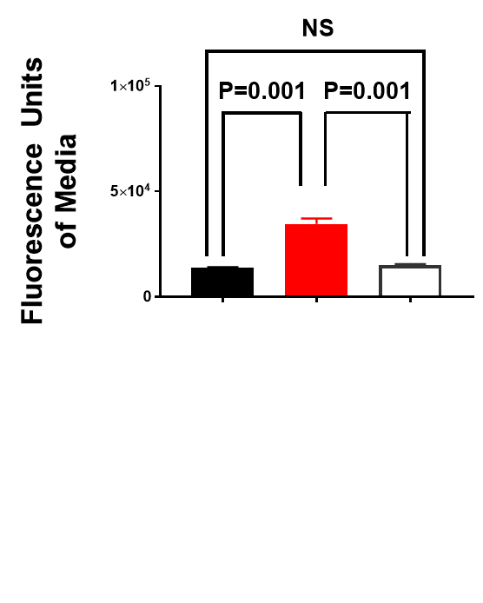


**(A)**

**(B)**

**(C)**

**(D)**

**PpIX-eGFP-FL**

**PpIX-FL Cell**

_**_ **- p<0.01**

_***_ **- p<0.001**

********

******

******

******

******

******

******

*******

**Supplemental Figure S13:** Effect of 24 h TTFields exposure (24h) on (A-B, E) Protoporphyrin IX fluorescence (PpIX-FL) and on (C-D) eGFP fluorescence (eGFP-FL) of U87-MG cells (A,B,C,D) and on the PpIX-FL of (E) conditioned media from the U87-MG cell culture. Cells were seeded onto 22mm glass coverslips, grown, and prepared for TTFields exposure according to the procedure described in **Supplemental Figure S1**. Cells were subjected to the conditions of: (1) standard, control tissue culture settings of 37^o^C, 95% O_2_, 5% CO_2_ and no exposure to TTFields, (2) 24 hours of TTFields exposure and (3) 24 hours of TTFields exposure followed by additional 24 hours of no TTFields exposure. All experimental conditions were done in triplicate and statistical analysis calculated via 2-way ANOVA. Both PpIX-FL and eGFP-FL imaging were acquired in the presence of 500 µg/ml 5-aminolevulinic acid (5-ALA) and recorded in an Ilumina II IVIS machine. eGFP fluorescence was read with Ex= 465 nm and Em= 510 nm while PpIX fluorescence data was collected with the parameters of Ex= 430 nm and Em= 680 nm. PpIX-FL intensity of media was read in a Tecan Infinite M200 plate reader. For the uptake experiments, cells were incubated in serum-free DMEM media in the presence of 500 $\mu$g/ml 5-ALA. PpIX-FL– Protoporphyrin IX fluorescence imaging, eGFP-FL-eGFP fluorescence, IVIS – Intravital Imaging System. Level of significance: ** represents p<0.01 and *** represents p<0.001.

**(D)**

**(C)**

**(D)**

**Supplemental Figure S14 (Dextran-FITC reversibility)**


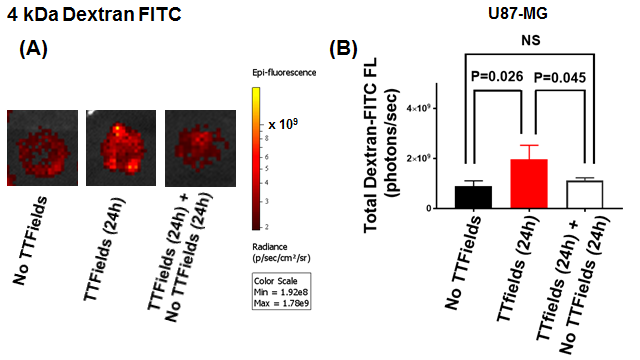


*****

*****

**_*_ - p<0.05**

**Supplemental Figure 14:** Panel of figures showing the reversibility of the effect of TTFields on the permeation of 4 kDa Dextran-FITC through the plasma membrane of U87-MG cancer cells. The extent of Dextran-FITC binding and likely uptake through the cell membrane was assessed by the level of FITC fluorescence of the unlabeled U87-MG cells (Excitation=465 nm, Emission=510 nm). Cells were subjected to the conditions of: (1) standard, control tissue culture settings of 37^o^C, 95% O_2_, 5% CO_2_ and no exposure to TTFields, (2) 24 hours of TTFields exposure and (3) 24 hours of TTFields exposure followed by additional 24 hours of no TTFields exposure. All experimental conditions were done in triplicate and statistical analysis calculated via 2-way ANOVA. NS represents no significant difference. (A) representative panel of fluorescent (FL) imaging scans as a function of the three aforementioned conditions (i.e. No TTFields, TTFields (24 h), TTFields (24 h) followed by no TTFields (24 h)) and (B) quantification of PpIX fluorescence intensity in (A). FITC = fluorescein isothiocyanate. * represents level of significance of p<0.05.

**Supplemental Figure S15 (Dextran-FITC reversibility data)**


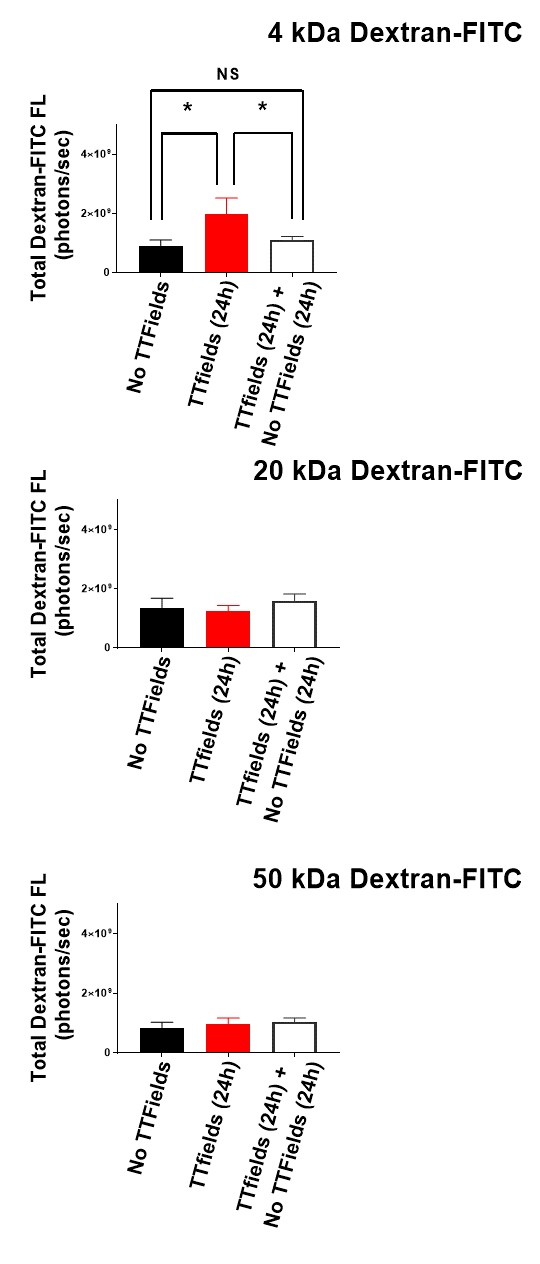


**Supplemental Figure S15:** Effect of 24 h TTFields exposure (24h) on Dextran-FITC binding (MW=4, 20 or 50 kDa) to cellular membranes of U87-MG cells. Cells were seeded onto 22 mm glass coverslips, grown, and prepared for TTFields exposure according to the procedure described in **Supplemental Figure S1**. Cells were subjected to the conditions of: (1) standard, control tissue culture settings of 37^o^C, 95% O_2_, 5% CO_2_ and no exposure to TTFields, (2) 24 hours of TTFields exposure and (3) 24 hours of TTFields exposure followed by additional 24 hours of no TTFields exposure. Cells were challenged with 1000 µg/ml of Dextran-FITC (MW=4, 20 and 50 kDa) in DMEM/10% FBS for the last two hours of the three experimental conditions before being washed twice with room temperature HBSS and the immersed in HBSS before fluorescence scanning. All experimental conditions were performed in triplicate and statistical analysis calculated via 2-way ANOVA. FITC fluorescence was acquired on the Lumina II Intra-vital Imaging System (IVIS) with Ex= 465 nm and Em= 510 nm. FITC – Fluorescein Isothiocyanate; HBSS-Hank’s Balanced Salt Solution; IVIS – Intravital Imaging System. * represents level of significance of p<0.05.

**Supplemental Figure S16 (U87-MG SEM Reversibility)**


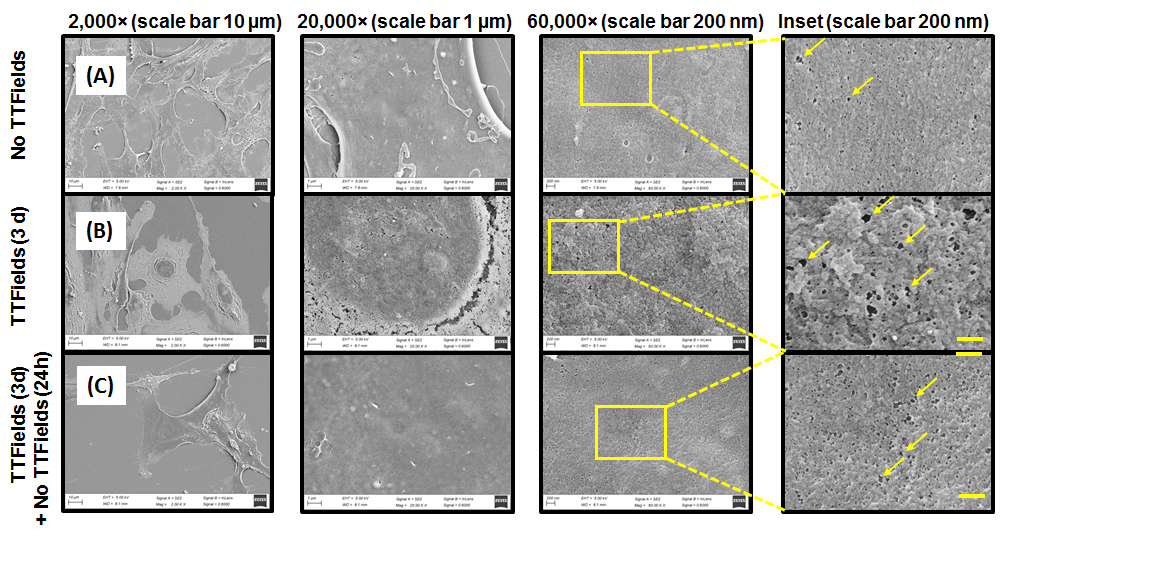


**Supplemental Figure S16:** Scanning electron micrographs of U87-MG/eGFP-fLuc cells seeded at 5,000 cells per 13 mm glass coverslips and then prepared for TTFields experiments under a protocol described in **Supplemental Figure S1**. Unless otherwise mentioned, cells were grown under standard tissue culture incubator conditions (37^o^C, 95% O_2_, 5% CO_2_). The experimental conditions are as follows: (A) cells that remained unexposed to TTFields, (B) cells that were exposed to TTFields (200 kHz) for 72 hours and (C) cells that have been exposed to TTFiels for 72 hours and then transferred to standard tissue culture conditions but no exposure to TTFields for 24 hours. At the end of each experiment, the coverslips were processed for scanning electron microscopy (SEM). Initially, the coverslips were fixed at room temperature with 4% paraformaldehyde/0.1M Na cacodylate buffer (pH 7.0) for 30 minutes. Then the solution was exchanged for 2% glutaraldehyde/0.1M Na cacodylate (pH 7.0) for another 30 minutes at room temperature. The samples were then stored on ice until the samples were submitted to the Cell Sciences Imaging Facility (for the low seeding density conditions) at Stanford and processed for SEM (**see Supplemental Figure S10**). Yellow arrows indicate holes appearing on cell membrane while yellow scale bar represents 200 nm


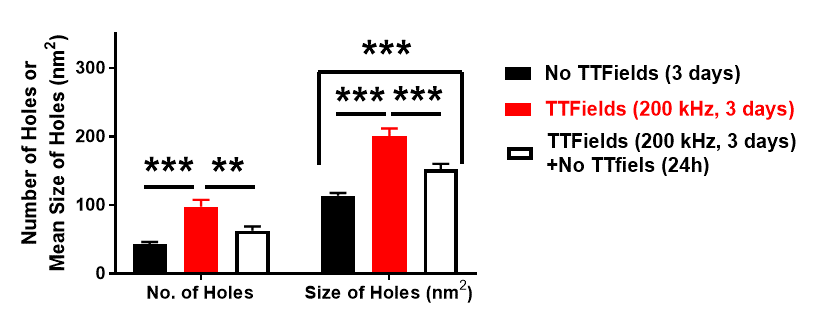
**Supplemental Figure S17 (U87-MG SEM Reversibility: Quantitation)**

**Supplemental Figure S17:** Quantitation of the number of holes and size of holes appearing on the cell surface of the U87-MG/eGFP-Luc samples shown in **Supplemental Figure S16.** Quantitation obtained through ROI analysis of holes on membrane surfaces as represented in **Supplemental Figures S16** and as described by **Supplemental Figure S10**. The above analysis was performed in a double blind manner. All experimental conditions were performed in triplicate and statistical analysis calculated via 2-way ANOVA with Bonferonni post-hoc correction. Data presented as mean + SEM of 15-24 ROI measurements per field of view. Level of significance: ** represents p<0.01 and *** represents p<0.001.

**Reference for Supplemental Figures:**

1. (5)-aminolevulinic_acid. <http://www.chemspider.com/Chemical-Structure.134.html>

2. coelenterazine_H. <http://www.chemspider.com/Chemical-Structure.2043421.html>

3. coelenterazine. <http://www.chemspider.com/Chemical-Structure.2728.html>.

4. isothiocyanate_(FITC)-dextran F. <https://www.sigmaaldrich.com/content/dam/sigma-aldrich/docs/Sigma/Product_Information_Sheet/1/fd250spis.pdf>

5. D-luciferin. <http://www.chemspider.com/Chemical-Structure.16735812.html>

6. Loening AM, Fenn TD, Gambhir SS. Crystal structures of the luciferase and green fluorescent protein from Renilla reniformis. J Mol Biol. 2007;374(4):1017-28.

7. Matthews JC, Hori K, Cormier MJ. Purification and properties of Renilla reniformis luciferase. Biochemistry. 1977;16(1):85-91.

8. Karkhanis YD, Cormier MJ. Isolation and properties of Renilla reniformis luciferase, a low molecular weight energy conversion enzyme. Biochemistry. 1971;10(2):317-26.

9. Protoporphyrin_IX. <http://www.chemspider.com/Chemical-Structure.10469486.html>; <https://pubs.acs.org/doi/pdf/10.1021/jp013155h>

10. Scolaro LM, Castriciano M, Romeo A, Patan S, Cefalì E, Allegrini M. Aggregation Behavior of Protoporphyrin IX in Aqueous Solutions:  Clear Evidence of Vesicle Formation. J Phys Chem B. 2002;106(10):2453–9.

11. Jazayeri FS, Amininasab M, Hosseinkhani S. Structural and dynamical insight into thermally induced functional inactivation of firefly luciferase. PLoS One. 2017;12(7):e0180667.

12. Conti E, Franks NP, Brick P. Crystal structure of firefly luciferase throws light on a superfamily of adenylate-forming enzymes. Structure. 1996;4(3):287-98.

13. (Ethidium_Bromide) ED. <http://www.chemspider.com/Chemical-Structure.14034.html>

14. Annexin V acAAV. <https://www.physiology.org/doi/abs/10.1152/physrev.00030.2001>; <https://www.sigmaaldrich.com/content/dam/sigma-aldrich/docs/Sigma/Product_Information_Sheet/a9210pis.pdf>.

15. Gerke V, Moss SE. Annexins: from structure to function. Physiol Rev. 2002;82(2):331-71.

16. Hink MA, Griep RA, Borst JW, van Hoek A, Eppink MH, Schots A, et al. Structural dynamics of green fluorescent protein alone and fused with a single chain Fv protein. J Biol Chem. 2000;275(23):17556-60.

17. Craggs TD. Green fluorescent protein: structure, folding and chromophore maturation. Chem Soc Rev. 2009;38(10):2865-75.

18. Pubchem. Molecular Weight of Firefly D-Luciferin 2017 [Available from: <https://pubchem.ncbi.nlm.nih.gov/compound/D-Luciferin>.

19. Pubchem. Molecular Weidght of Coelentrazine 2017 [Available from: <https://pubchem.ncbi.nlm.nih.gov/compound/2762722>.

20. van Engeland M, Nieland LJ, Ramaekers FC, Schutte B, Reutelingsperger CP. Annexin V-affinity assay: a review on an apoptosis detection system based on phosphatidylserine exposure. Cytometry. 1998;31(1):1-9.

21. Chang E, Pohling C, Natarajan A, Witney TH, Kaur J, Xu L, et al. AshwaMAX and Withaferin A inhibits gliomas in cellular and murine orthotopic models. J Neurooncol. 2016;126(2):253-64.

22. Ram Z, Kim CY, Nicholas GA, Toms SA. ACTR-27. Compliance and Treatment Duration Predict Survival in a Phase 3 EF-14 Trial of Tumor Treating Fields with Temozolomide in Patients with Newly Diagnosed Glioblastoma. Neuro-Oncology. 2017;19(suppl_6):vi6–vi7.
